# Evogliptin prevents ceramide-induced pyroptosis during calcification via modulation of NLRP3/GSDM-D mediated pathway in Vascular Smooth Muscle Cells

**DOI:** 10.1371/journal.pone.0337200

**Published:** 2025-12-03

**Authors:** Razia Rashid Rahil, Salman Shamas, Nissar Ahmad Wani, Neha Nanda, Sheikh Fayaz Ahmad, Sabry Mohamed Attia, Abid Hamid, Mohammad Afzal Zargar, Owais Mohmad Bhat

**Affiliations:** 1 Department of Biotechnology, School of Life Sciences, Central University of Kashmir, Ganderbal, India; 2 Koch Institute for Integrative Cancer Research at MIT, Cambridge, Massachusetts, United States of America; 3 Department of Pharmacology and Toxicology, College of Pharmacy, King Saud University, Riyadh, Saudi Arabia; Noorda College of Osteopathic Medicine, UNITED STATES OF AMERICA

## Abstract

Evogliptin, an anti-diabetic drug had positive impact on various cardiovascular events including inflammation and vascular calcification (VC), an active process driven by vascular smooth muscle cell (VSMC) phenotypic transition. Sphingolipids such as ceramide (CER) mediates inflammation and VC in the vascular tissue. We investigated whether evogliptin ameliorate phenotypic transition and pyroptosis in VSMCs as underlying cause of VC. In cultured VSMCs, isolated from the aorta of (C57/BL6) mouse, we observed more severe calcification with prior treatment of CER in P_i_-treated VSMCs as detected by Alizarin Red Staining. Prior CER- stimulation led to a marked upregulation of osteogenic markers such as RUNX2, OPN, BMP2 and decreased contractile markers SM22-α and α- SMA in P_i_-treated VSMCs as compared to control cells. In addition, increased expression of pyroptotic markers such as NLRP3, GSDM-D, IL-1*β*, IL-18, and LDH release was observed with prior treatment of CER in P_i_-treated VSMCs as compared to control cells. Furthermore, MCC950 (NLRP3 inhibitor), disulfiram (GSDM-D inhibitor) and evogliptin significantly downregulated osteogenic and pyroptotic markers including LDH release in both P_i_‐induced only and CER + P_i_-treated VSMCs. Moreover, GW4869 (SMase inhibitor) and evogliptin significantly reduced SMase activity in sphingomyelin (SM)-induced VSMCs as compared to both P_i_ and SM only-treated groups. Also, the cleavage efficiency of GSDM-D was high in P_i_ and CER + P_i_ groups which was reduced with prior treatment of evogliptin. Hence, our data demonstrate that evogliptin alleviates VC by blocking phenotypic transition and associated pyroptosis via modulation of NLRP3/GSDM-D mediated pathway in CER-induced VSMCs.

## Introduction

VC is described as anomalous deposits of mineral matrix within the vessel wall [1,2]. Several studies have demonstrated that calcification stimulators result in increased expression of bone formation-related transcription factors such as Msx2, Runx2, and Osterix in VSMCs [3,4]. There are two major mechanisms underlying the pathophysiology of VC: the loss of inhibitors of calcification, and the dysregulation of phosphorus (and calcium) homeostasis [5]. Several other secondary mechanisms include the phenotypic transition of VSMCs to an osteochondrogenic phenotype, the degradation of extracellular matrix, circulating nucleation complexes and apoptosis [5,6]. Studies have revealed that increase in serum inorganic phosphate or hyperphosphatemia, is a substantial risk factor for VC in both hemodialysis patients and the general population [7,8].

Sphingolipids play an important role in the pathology of various diseases such as cancer, obesity, diabetes, neurodegenerative and cardiovascular diseases. Several studies demonstrated that sphingolipids such as ceramide (CER) enhanced aortic valve stenosis, and result in phenotypic transition including small extracellular vesicles (sEVs) secretion in arterial SMCs leading to arterial stiffness [9,10]. In the present study we investigated whether CER enhanced VC via NLRP3 activation and inflammatory cell death of VSMCs. Literature reports that inhibition of NLRP3/NF-κB signaling pathway attenuates calcification in uremic rat, VSMCs and human aortic valve interstitial cells (VIC) [11–13]. Besides, NLRP3 activation caused phenotypic transition in high glucose treated VSMCs while as inhibition of NLRP3 ameliorates VC and insulin resistance in β-cells of rats fed on high fat/glucose diet, also reduced plaque size in ApoE-/- mice fed on high-fat diet [14–16]. Recently, a study found that increased ROS production and NLRP3 activation leads to VC and pyroptosis in β-glycerophosphate treated VSMCs in chronic kidney disease (CKD) [17].

Pyroptosis, a regulated type of inflammatory cell death, induced by a variety of stimuli such as DAMPs, PAMPs, uric acid crystals, toxins, phosphate, ROS, lipids such as palmitic acid, ox-LDL, etc., and lead to NLRP3 inflammasome activation, release of inflammatory cytokines and cell death [18–20]. The activation of the NLRP3 inflammasome by calcium phosphate crystals occurs in the early stages of cultured cells, implies that the NLRP3 signaling is an acute response to calcium phosphate or mineral deposition during calcification [21]. Also, inhibition of GSDM-D pore formation in both canonical and non-canonical inflammasome pathways by disulfiram, a Gasdermin-D (GSDM-D) inhibitor, leads to inhibition of pyroptosis under *in-vitro* and *in-vivo* conditions [22]. Hence, we aim to investigate whether NLRP3/GSDM-D pathway contributes to CER-induced pyroptosis in VSMCs during VC.

Literature cites, type-II diabetes is closely associated with VC, and several drugs used for type-II diabetes can pave way in regulating the vascular remodeling and may be exploited as the therapeutics for VC [23,24]. Evogliptin, a DPP-4 inhibitor, augmented Nrf2 and HO1 (a target gene of Nrf2) expression, also reduced inflammation of liver via autophagy and inhibition of liver fibrotic signaling in primary hepatocytes [25]. This study suggested that evogliptin may be beneficial for liver inflammation and fibrosis in diabetic patients. A study under *in-vitro* and *in-vivo* conditions reported that evogliptin ameliorates the mineralization of the primary human valvular interstitial cells (VICs), calcific deposition and proinflammatory cytokine expression in eNOS-deficient mice, also supressed the Vitamin D2-driven fibrosis in high-cholesterol diet fed rabbit model [26]. Increased expression of DPP-4 was observed during endothelial dysfunction in the aortic valve which leads to degradation of insulin-like growth factor-1 (IGF-1) and increases osteogenic differentiation of vascular interstitial cells [27]. As evogliptin displays high specific accumulation in cardiac tissue [28], hence we here evaluated its therapeutic potency for ameliorating phenotypic transition an underlying mechanism which contributes to VC in VSMCs. Therefore, in the present study, firstly we investigated whether NLRP3/GSDM-D pathway contributes to CER-induced pyroptosis in VSMCs during VC. Secondly, we elucidated the role of evogliptin in modulating Sphingomyelinase (SMase) activity and CER-induced pyroptosis which contributes to pathogenesis of VC via NLRP3/GSDM-D-mediated pathway in VSMCs.

## Methodology

### Primary culture of VSMCs and Alizarin Red S staining

Vascular Smooth Muscle Cells (VSMCs) were isolated by Collagenase II degradation method from the aorta of (C57/BL6) mice [29]. VSMCs were cultured in Dulbecco’s Modified Eagle Medium (DMEM) with 10% Fetal Bovine Serum (FBS) and 1% penicillin streptomycin in humidified 100% air at 37°C with 5% CO_2_. Confluent cells were treated with high phosphate (P_i_) (3 mmol/L) [30] and Ceramide (5μm/L) [31], and osteogenic medium was changed every two days for 14 days to develop *in vitro* calcification model. VSMCs were rinsed with PBS, fixed with 10% formalin for 30 minutes. Then, cells were rinsed three times with distilled water and incubated with 1% Alizarin Red Stain for 15 minutes. Finally, cells were washed with distilled water and visualized under Bright Field Microscope. Positive staining is represented as a red/orange colour while the controls were faintly yellow. For quantification, cells were incubated with the solution of 20% methanol and 10% acetic acid in water for 15 minutes and absorbance was measured at 405nm. VSMCs were also treated with pharmacological inhibitor of NLRP3 (MCC950) (7μm/L) [32], GSDM-D (Disulfiram) (10μm/L) [22] and evogliptin (10μm/L) [26] for 24 hours.

### Scanning Electron Microscopy (SEM) and Energy Dispersive X-ray Spectroscopy (EDXS) analysis

For this, the cells were grown on glass substrate. Post treatments, cells were fixed with 3.7% glutaraldehyde and then subjected to dehydration with ascending sequence of ethanol (40%, 60%, 80%, 98%), incubated at room temperature overnight. The next day, the samples were subjected to gold-palladium sputtering and finally analysed using SEM. A Zeiss Supra 55 VP FEG-SEM with a Gemini column was used, complemented by additional micrographs of the VSMCs. Mineral deposition including calcium and phosphate was measured using EDXS analysis (Supra 55VP, Carl Zeiss). Samples were prepared as above, EDX spectra were acquired from samples at 8 kV while mappings were acquired at 5 kV using an Oxford Instruments XMax 150 SDD detector in combination with the AZtec software suite.

### Calcium assay

The calcium content in cells was quantified using calcium assay kit (Calcium Colorimetric Assay Cat. no. MAK022) as per manufacturers protocol. The protein content of the cells was quantified using the “Micro BCA Protein Assay Kit”. The ratio of calcium content to protein content (*in vitro*) was evaluated and statistically checked. The calcium concentration was measured as below-


Calcium concentration = (Absorbance of sample/Absorbance of standard) ×Standard concentration.

### Quantitative real time PCR analysis

The mRNA expression of VSMC phenotypic switch markers (SM22α, α SMA, BMP 2, OPN, RUNX2) and pyroptotic markers (NLRP3, GSDM-D, IL-1β and IL-18) were analysed using gene specific primers and GAPDH was used as an internal control. The cells were collected by centrifugation, and RNA was extracted using TRIzol/chloroform method (Sigma Aldrich Corporation). The concentration and purity of total RNA was determined by measuring the optical density at 260nm and 280nm using NanoDrop spectrophotometer. The integrity of RNA was assessed by gel electrophoresis. RNA was treated with DNase I and used for the reverse transcription using the high-capacity cDNA reverse transcription kit (PrimeScript™ Ist strand cDNA Synthesis Kit (TaKaRa Bio USA, Inc.). A reverse-transcription reaction system (with a final volume 20 µL) containing 1 µL of 50 µM Random hexamers, 1 µL of 10mM deoxyribonucleoside triphosphate mixture and 1 µg RNA was incubated for 5 min at 65ºC and then on ice. After incubation, 4 µL of 5X reaction buffer, 0.5 µL of RNase inhibitor and 1 µL of reverse transcriptase were added to the reaction mixture. Final volume was adjusted to 20 µL with nuclease free water. Reverse transcription was done at 42ºC for 1 hour. Reaction was stopped at 72ºC for 10 min followed by cooling on ice, finally cDNA was stored at −20ºC.

qRT-PCR using TB Green Premix Ex Taq II (TaKaRa Bio USA, Inc.) was performed on CFX96™ Real-time System (BioRad). The amplification reaction consisted 10 µL of 2X SYBR Premix ExTaq II, and 1 µL cDNA template qRT-PCR was carried out in duplicate for each sample using the following parameters: I cycle at 95ºCfor 10 min followed by 39 cycles at 95ºC for 15 sec (denaturation), melting temperature of primers specific for particular gene for 15 sec (annealing), and 72ºC for 30 sec (extension). GAPDH was used as the internal control. The stability of normalization control was checked by RefFinder, a comprehensive web tool developed for evaluating and screening reference genes. 2^-ΔΔCt^ method was used for the analysis of qRT-PCR results [33]. The primer sequences used are listed below:

**Table:** 

Gene	Sense (5′-3′)	Antisense (5′-3′)
SM22α	TCCAGGTCTGGCTGAAGAATGG	CTGCTCCATCTGCTTGAAGACC
α SMA	CACTATTGGCAACGAGCGC	CCAATGAAGGAAGGCTGGAA
RUNX2	CCCAGTATGAGAGTAGGTGTCC	GGGTAAGACTGGTCATAGGACC
BMP 2	TGTATCGCAGGCACTCAGGTCA	CCACTCGTTTCTGGTAGTTCTTC
OPN	CGAGGTGATAGTGTGGTTTATGG	GCACCATTCAACTCCTCGCTTTC
GSDM-D	GTGTGTCAACCTGTCTATCAAGG	CATGGCATCGTAGAAGTGGAAG
NLRP3	ATTACCCGCCCGAGAAAGG	TCGCAGCAAAGATCCACACAG
IL-1β	CCACAGACCTTCCAGGGAATG	GTGCAGTTCAGTGATCGTACAGG
IL-18	GATAGCCAGCCTAGAGGTATG	CCTTGATGTTATCAGGAGGATTC
GAPDH	GTCTCCTCTGACTTCAACAGCG	ACCACCCTGTTGCTGTAGCCAA

### Western blot analysis

Briefly, the cells were lysed with RIPA buffer containing protease inhibitor cocktail and micro-centrifuged. Proteins in the supernatant were extracted, then equal amount of protein was resolved on 12% poly acryl amide gels and then transferred to PVDF membrane using Trans-Blot turbo transfer system. The membranes were blocked and incubated with anti-SM22- α, anti-OPN, anti-RUNX-2, anti-GSDM-D and anti-NLRP3 primary antibodies at 4°C overnight. Next day, the membranes were incubated with secondary antibodies diluted in 0.1% TBST at room temperature for 2 hours. The blots were developed with ECL and protein bands were visualized under ChemiDoc Imaging System. GAPDH was used as an internal control [33].

### Lactate Dehydrogenase assay (LDH)

Cell death was determined according to the manufacturer’s protocol using the LDH assay kit. Using a plate-reading spectrophotometer, optical density values were measured at 492 nm and 680 nm to account for any background absorbance values. The percentage of cytotoxicity was determined as follows for each absorbance:


$$\begin{array}{cc} \%\text{ cytotoxicity }= & \;(\text{Compound treated LDH activity }-\text{ Spontaneous LDH activity}/\text{Maximum LDH activity }\\ & -\text{ Spontaneous LDH activity})\times \text{100}\% \end{array}$$

The cytotoxicity obtained at 680 nm were subtracted from the 492 nm to account for back ground absorbance.

### *In-silico* analysis

Molecular docking studies were performed to check the binding sites and interactions of evogliptin with Sphingomyelinase (SMase) enzyme. The three-dimensional structure of acid sphingomyelinase (SMase) was obtained from the Protein Data Bank (PDB ID: 1CQD). The protein was prepared for docking by removing water molecules, adding polar hydrogens, and optimizing with the AutoDockTools 1.5.7 interface/ Schrödinger-PyMOL. The chemical structure of Evogliptin was retrieved from PubChem (CID: 10001686) and energy-minimized using the MMFF94 force field. Molecular docking analysis was performed using AutoDock Vina to predict potential binding sites of Evogliptin on SMase. A blind docking approach was applied with a grid box size of 24 × 24 × 24 Å³, centred at coordinates (24, 24, 24) to cover the active and nearby regions. Among the identified binding pockets, cluster C1 exhibited the most favourable interaction with a Vina score of −9.0 kcal/mol and a cavity volume of 2113 Å³. Residues involved in ligand binding included A451, S508, L450, N448, R474, Y511, P550 and T548, forming hydrogen bonds and hydrophobic interactions that stabilize the Evogliptin–SMase complex.

### Sphingomyelinase (SMase) assay

Briefly, VSMCs were seeded in 6-well plates at a density of 1 × 10^5^ cells/ cm^2^ and cultured in osteogenic medium supplemented with or without different treatment groups. Cells were washed with PBS and harvested by lysis buffer in presence of protease inhibitor cocktail. BCA protein assay was used to quantify cellular protein concentration. 50 µL of cell lysates and standards were taken in 96 well plate, diluted with 50 µL of the working solution. The plate was subjected to horizontal shaking followed by incubation for 2 hours at 37°C. Then, 50 µL of the Sphingomyelinase Assay Mixture was added to each sample, standard, and blank, incubated for 1 hour at 37°C. The absorbance was measured by a plate reader (EPOCH, BioTEK) at 655nm and calculated as U/ml compared to the standards.

### Statistical analysis

Data were analysed using the GraphPad prism software. Results are presented as mean ± standard deviation (SD). Statistical analysis was performed using unpaired two-tailed Student’s *t* test for comparisons between two groups, and ANOVA followed by a Holm–Sidak test was used to test for differences involving more than two groups. For all statistical tests, a difference was considered significant at *P* < 0.05.

## Results

### Vascular calcification and phenotypic switch in P_i_*‐*treated VSMCs

**Fig 1:**
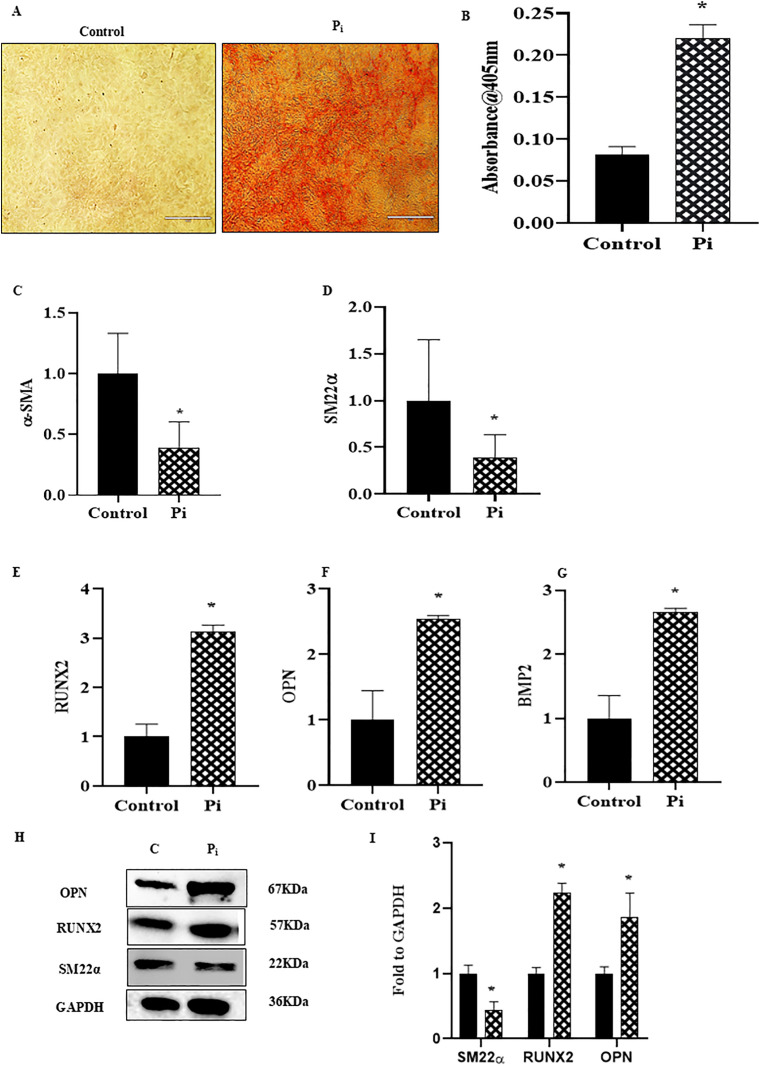
P_i_‐treatment aggravates calcification and induces phenotypic transition of VSMCs. (A) Representative images showed calcium deposition(red) in P_i_-induced VSMCs. (B) Summarized bar graph showed P_i_ treatment significantly increased calcium deposition. Bar graphs depicted significant decrease in mRNA expression of (C) α -SMA (D) SM22α and increased (E) RUNX2 (F) OPN and (G) BMP2. (H-I) Representative western blots and summarized bar graph showed significant decrease in protein expression of SM22α and increased RUNX2 in P_i_-induced VSMCs. (n = 3). Results are presented as mean ±SD. Phosphate: P_i_. *(P < 0.05) significant vs. control group; ^$^(P < 0.05) significant vs. P_i_ group by students t-test.

Cells were subjected to osteogenic medium for two weeks and osteogenic media was changed on alternate days. We observed significantly increased calcification in P_i_‐induced VSMCs as compared to control as detected by Alizarin Red S staining. The summarized data is depicted in the Fig 1(A) and 1(B). Furthermore, cells were subjected to osteogenic medium for 24 hours. Using real‐time PCR, we found mRNA expression of contractile markers SM22α and α-SMA were significantly decreased while osteogenic markers like RUNX2, BMP2 and OPN were increased in the P_i_‐induced VSMCs (Fig 1C–1G). These results were in consistence with the western blot results which showed significant decrease in SM22α and increase in the RUNX2 and OPN expression in P_i_-induced VSMCs (Fig 1H–1I).

### Ceramide enhanced vascular calcification and phenotype change in P_i_*‐*treated VSMCs

**Fig 2:**
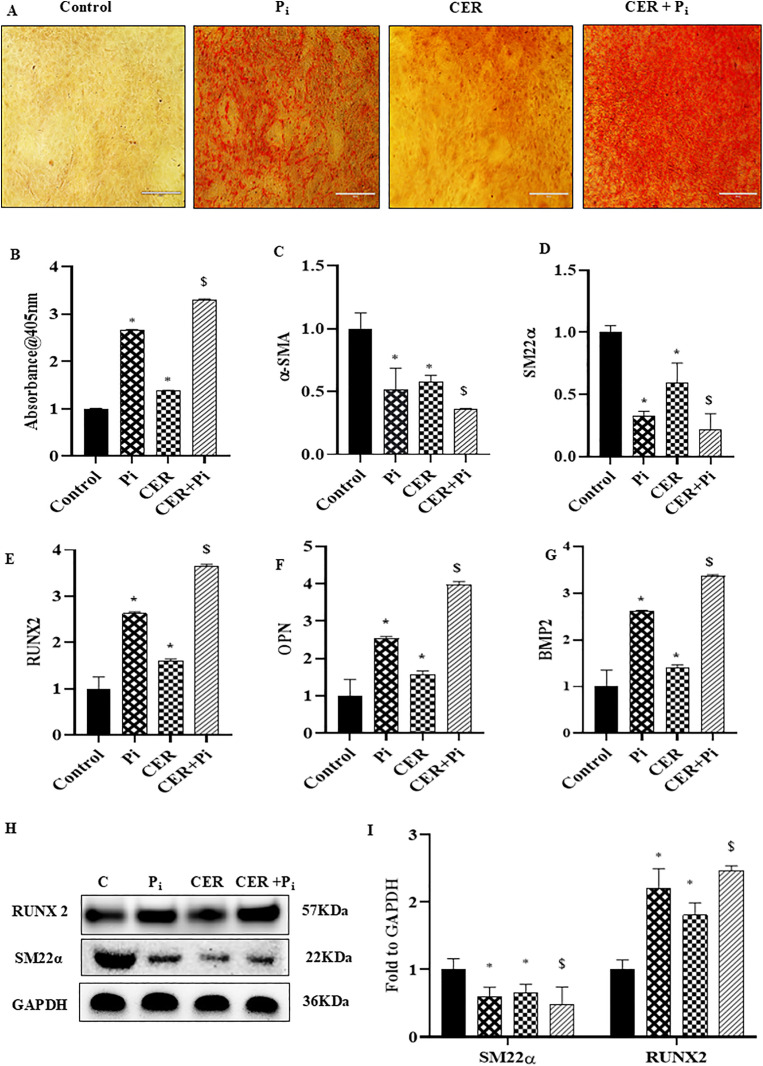
Effects of CER on vascular calcification and phenotypic change in P_i_*‐*treated VSMCs. (A) Representative images showed calcium deposition (red) in CER treated P_i_-induced VSMCs. (B) Summarized bar graph showed CER treatment significantly increased calcium deposition in P_i_-induced VSMCs. Bar graphs depicted significant decrease in mRNA expression of (C) α -SMA (D) SM22α and increased (E) RUNX2 (F) OPN and (G) BMP2 in CER treated P_i_-induced VSMCs. (H-I) Representative western blots and summarized bar graph showed significant decrease in protein expression of SM22α and increase in RUNX2 in CER treated P_i_-induced VSMCs. (n = 3). Results are presented as mean ± SD. Ceramide: CER. *(P < 0.05) significant vs. control group; ^$^(P < 0.05) significant vs. P_i_ group by One way ANOVA followed by Tukey’s test.

**Fig 3:**
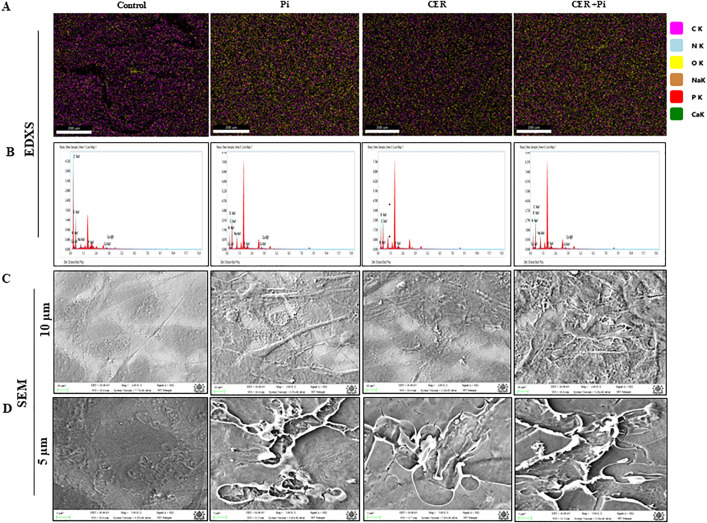
CER enhances mineral deposition and pyroptosis in Pi*‐*treated VSMCs. (A-B) Energy-Dispersive X-ray Spectroscopy (EDXS) analysis showed that among elements calcium concentration was relatively higher in P_i_ and CER treated Pi-induced VSMCs. (C-D) Representative scanning electron micrographs of VSMCs showed enlarged cell morphology and bubble-like extensions representing pyroptosis in P_i_ as well as CER treated P_i_-induced VSMCs. Ceramide: CER. (Scale bar, 100 nm) (C)10µm scale (D)5µm scale.

Here, we tested whether CER treatment enhanced VC in P_i_‐induced VSMCs. As shown in Fig 2(A) and 2(B), calcium deposition indicated by Red‐stained nodules were significantly increased with CER-treatment in P_i_*‐*induced VSMCs. Using real‐time PCR, we observed that CER only treatment significantly upregulated mRNA expression of RUNX2, OPN and BMP2 whereas SM22α and α-SMA were significantly decreased as compared to control. Moreover, CER treatment markedly enhanced expression of osteogenic markers in P_i_‐induced VSMCs as shown in Fig 2(C)–2(G). Also, western blot analysis showed that CER treatment aided osteogenic shift in P_i_‐induced VSMCs indicated with significantly increased protein expression of RUNX2 and decreased SM22α expression (Fig 2H–2I). Furthermore, we performed energy dispersive X-ray spectroscopy (EDXS) to all treatment groups. EDXS analysis showed that both P_i_ and CER + P_i_ treatment groups have chemistry typical of calcium and phosphates. Literature reports that ideal hydroxyapatite has Ca/P ratio of 1.67 [34].Our results showed Ca/P ratio of 1.62 and 1.85 in P_i_ and CER + P_i_ treated groups. As shown in Fig 3(A) and 3(B), EDX-mappings over the region of interest shows high calcium content in the VSMCs treated with P_i_ and CER + P_i_, with organic content distributed homogenously. The samples also contain magnesium, sodium, phosphorus, and oxygen in addition to calcium. Together, these results indicate that sphingolipid-ceramide contributes to the phenotypic transition, enhanced the mineralization and calcification in VSMCs.

### Ceramide augmented expression of pyroptotic markers in P_i_*‐*treated VSMCs

**Fig 4:**
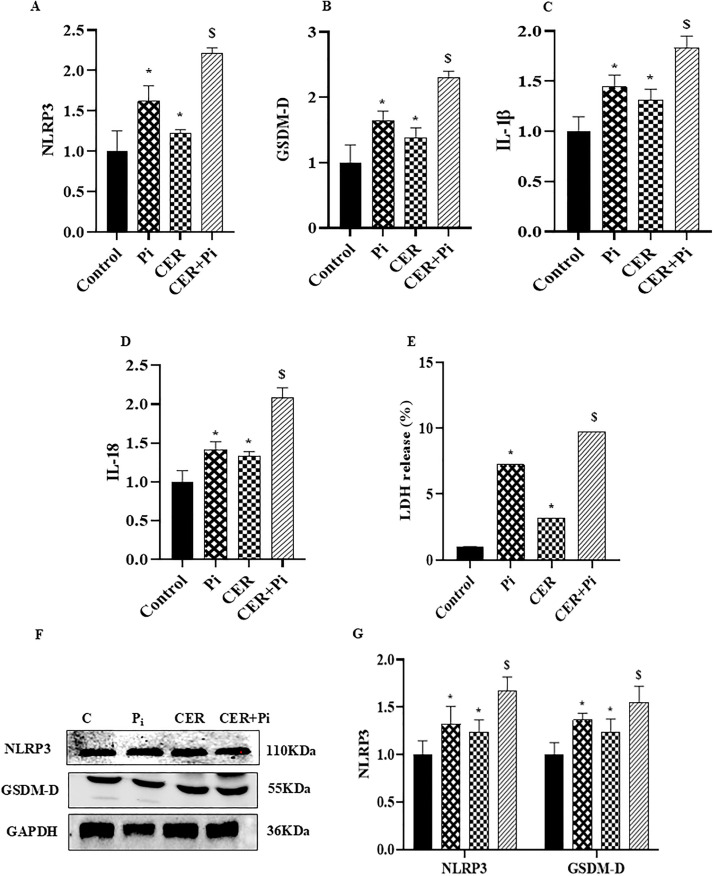
Effects of CER on expression of pyroptotic markers in P_i_*‐*treated VSMCs. (A-D) Summarized bar graphs showed significant increase in mRNA expression of pyroptotic markers (A) NLRP3 (B) GSDM-D, proinflammatory cytokines (C) IL-1ß (D)IL-18 and (E) increased Lactate dehydrogenase (LDH) release in CER-treated P_i_*‐*induced VSMCs. (F-G) Representative western blots and summarized bar graph showed significant increase in protein expression of NLRP3 and GSDM-D in CER-treated P_i_*‐*induced VSMCs. (n = 3). Results are presented as mean ±SD. Ceramide: CER. *(P < 0.05) significant vs. control group; ^$^(P < 0.05) significant vs. P_i_ group by One way ANOVA followed by Tukey’s test.

Recently, several studies reported that pyroptosis, an inflammatory cell death is associated with pathogenesis of VC in atherosclerosis [17,35]. Therefore, in the present study we tried to explore whether sphingolipid such as CER aided pyroptosis during VC. Post P_i_ and CER treatment for 24 hours, pyroptotic VSMCs were characterized by SEM analysis. We found that pyroptotic VSMCs were morphologically enlarged with protrusions, and developed bubble-like extensions prior to cell rupture as shown in Fig 3(C) and 3(D). Furthermore, Fig 4(A)–4(D) showed that CER only as well as CER + P_i_ significantly augmented mRNA expression of pyroptotic markers such as NLRP3 and GSDM-D, also increased mRNA expression of pro-inflammatory cytokines such as IL-1β and IL-18 including LDH release as compared to control group (Fig 4E) indicated that CER enhanced such phenomenon in P_i_‐induced VSMCs. Furthermore, CER treatment led to much more significant increase in the protein expression of pyroptotic markers NLRP3 and GSDM-D as shown in Fig 4(F) and 4(G). These findings indicate that sphingolipids such as CER promotes pyroptotic process in P_i_‐induced VSMCs during VC.

### Inhibition of NLRP3 blocked ceramide-induced pyroptosis and phenotypic transition in P_i_*‐*treated VSMCs

**Fig 5:**
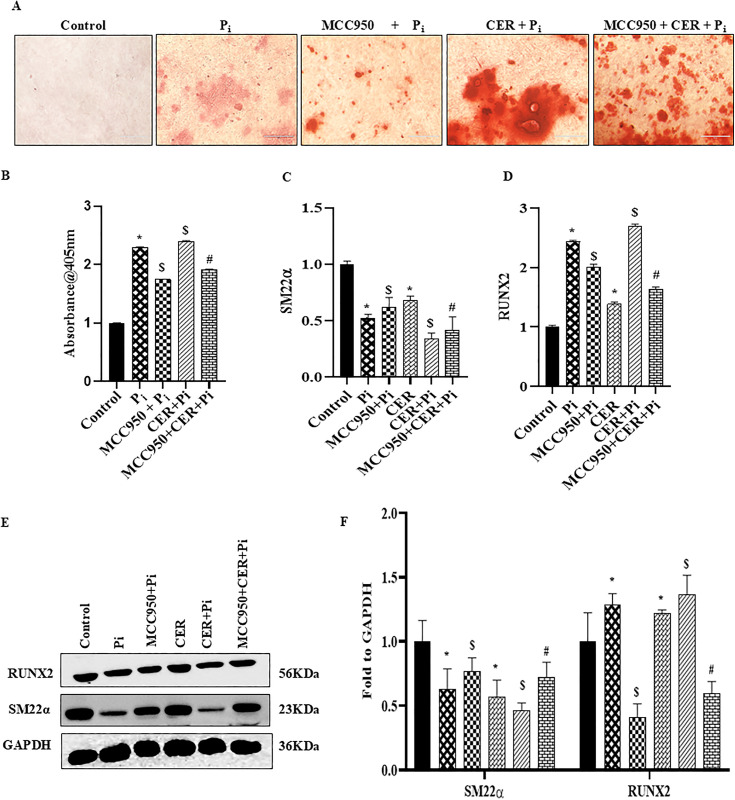
Effect of NLRP3 inhibition on expression of phenotypic transition markers in CER treated VSMCs during Vascular Calcification. (A) Representative images showed calcium deposition (red). (B) Summarized bar graph showed MCC950 significantly decreased calcium deposition in CER treated P_i_-induced VSMCs. Bar graphs depicted prior treatment with MCC950 significantly increased mRNA expression of (C) SM22α and decreased (D) RUNX2 in CER-treated P_i_*‐*induced VSMCs. (E-F) Representative western blots and summarized bar graph showed MCC950 significantly increased protein expression of SM22α and decreased RUNX2 in CER-treated P_i_*‐*induced VSMCs. (n = 3-4). Results are presented as mean ±SD. Ceramide: CER. *(P < 0.05) significant vs. control group; ^$^(P < 0.05) significant vs. P_i_ group; ^#^(P < 0.05) significant vs. CER + P_i_ group by One way ANOVA followed by Tukey’s test.

**Fig 6:**
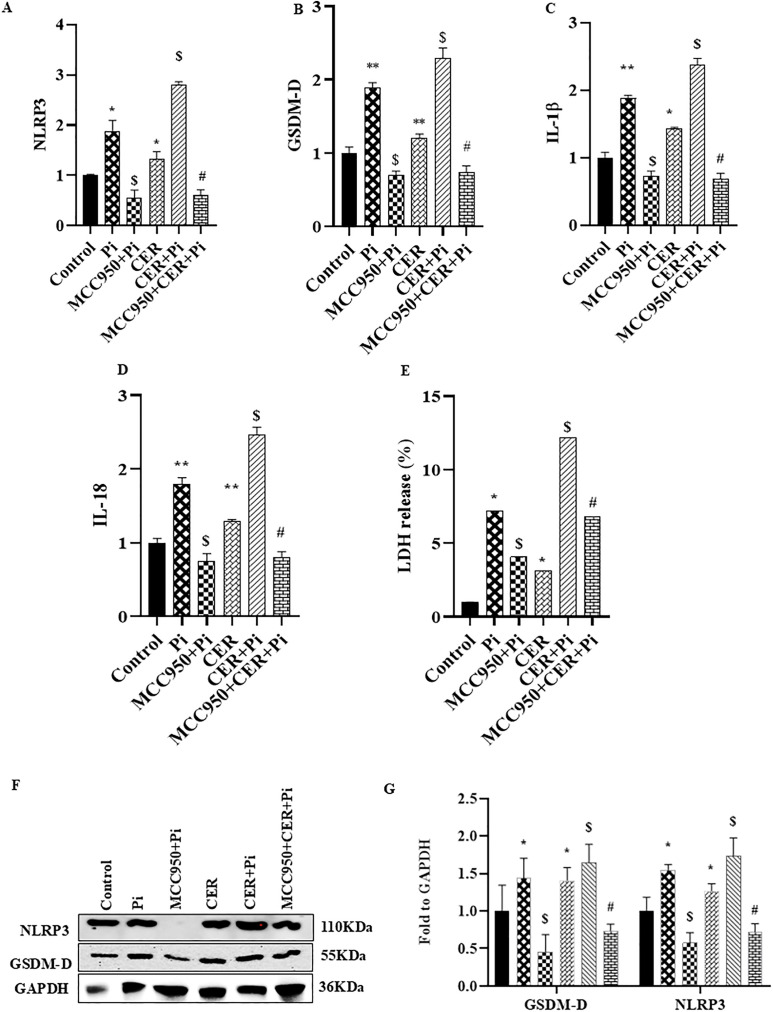
Effect of NLRP3 inhibition on CER-induced pyroptosis in P_i_*‐*treated VSMCs. Summarized bar graphs showed MCC950 significantly decreased mRNA expression of pyroptotic markers (A) NLRP3 (B) GSDM-D, proinflammatory cytokines (C) IL-1β (D) IL-18, and (E) Lactate dehydrogenase (LDH) release in CER treated P_i_‐induced VSMCs. (F-G) Representative western blots and summarized bar graph showed MCC950 significantly decreased protein expression of NLRP3 and GSDM-D in CER-treated P_i_*‐*induced VSMCs. (n = 3–4). Results are presented as mean ±SD. Ceramide: CER. *(P < 0.05) significant vs. control group; ^$^(P < 0.05) significant vs. P_i_ group; ^#^(P < 0.05) significant vs. CER + P_i_ group by One way ANOVA followed by Tukey’s test.

Next, we explored whether NLRP3 Inflammasome is associated with the CER-induced pyroptosis and its impact on VC. Prior treatment of MCC950, a specific inhibitor of NLRP3 significantly decreased VC in both P_i_‐induced and CER treated VSMCs as detected by Alizarin Red S staining in Fig 5(A), the summarized data is depicted in the Fig 5(B). Also, the mRNA and protein expression of phenotypic switch marker such as RUNX2 was significantly downregulated whereas SM22α was upregulated with MCC950 treatment in both P_i_ and CER + P_i_ treated groups as shown in Fig 5(C) and 5(D). Moreover, we observed that MCC950 treatment significantly decreased mRNA expression of pyroptotic markers such as NLRP3, GSDM-D, IL-1β and IL-18 in P_i_ and P_i_ + CER treated VSMCs (Fig 6A–6D). As shown in Fig 6(E) with prior MCC950 treatment, LDH release was significantly decreased in P_i_ and P_i_ + CER groups. Our western blot results are in tune with mRNA expression of GSDM-D and NLRP3 as shown in Fig 6(F) and 6(G). Hence, we can infer that inhibition of NLRP3 inflammasome prevents phenotypic transition of VSMCs from contractile to synthetic and also blocked CER-induced pyroptosis during VC.

### Inhibition of Gasdermin-D (GSDM-D) blocked ceramide-induced pyroptosis and phenotypic transition in P_i_*‐*treated VSMCs

**Fig 7:**
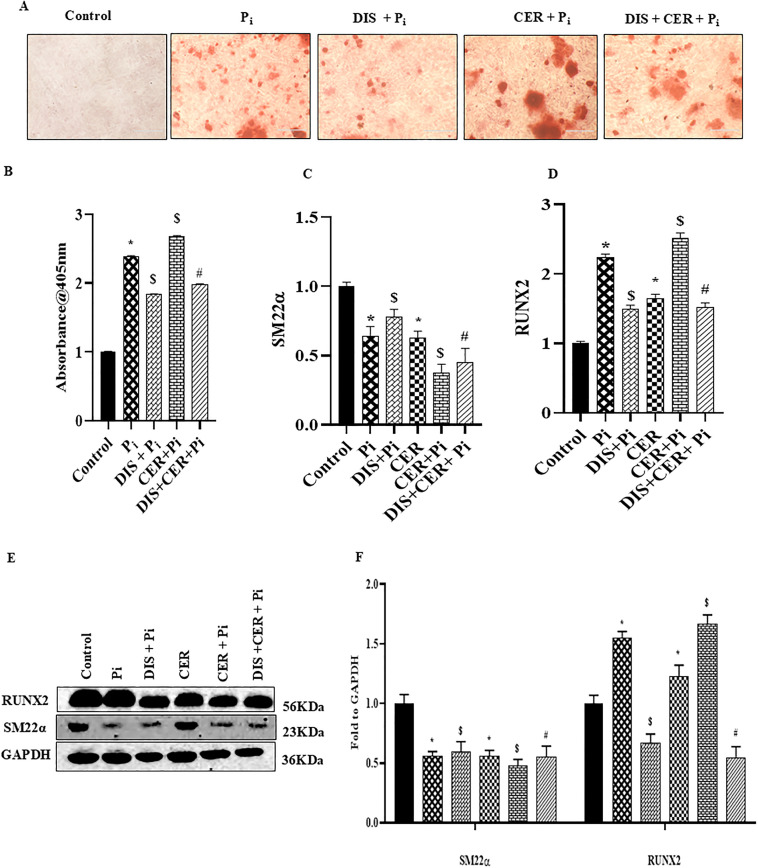
Effect of Gasdermin-D inhibition on expression of phenotypic transition markers in CER treated VSMCs during Vascular Calcification. (A) Representative images showed calcium deposition (red). (B) Summarized bar graph showed DIS significantly decreased calcium deposition in CER treated P_i_-induced VSMCs. Bar graphs depicted DIS increased mRNA expression of (C) SM22α and decreased (D) RUNX2 in CER-treated P_i_*‐*induced VSMCs. (E-F) Representative western blots and summarized bar graph showed DIS significantly increased protein expression of SM22α and decreased RUNX2 in CER treated P_i_-induced VSMCs. (n = 3–4). Results are presented as mean ± SD. Ceramide: CER, Disulfiram: DIS. *(P < 0.05) significant vs. control group; ^$^(P < 0.05) significant vs. P_i_ group; ^#^(P < 0.05) significant vs. CER + P_i_ group by One way ANOVA followed by Tukey’s test.

**Fig 8:**
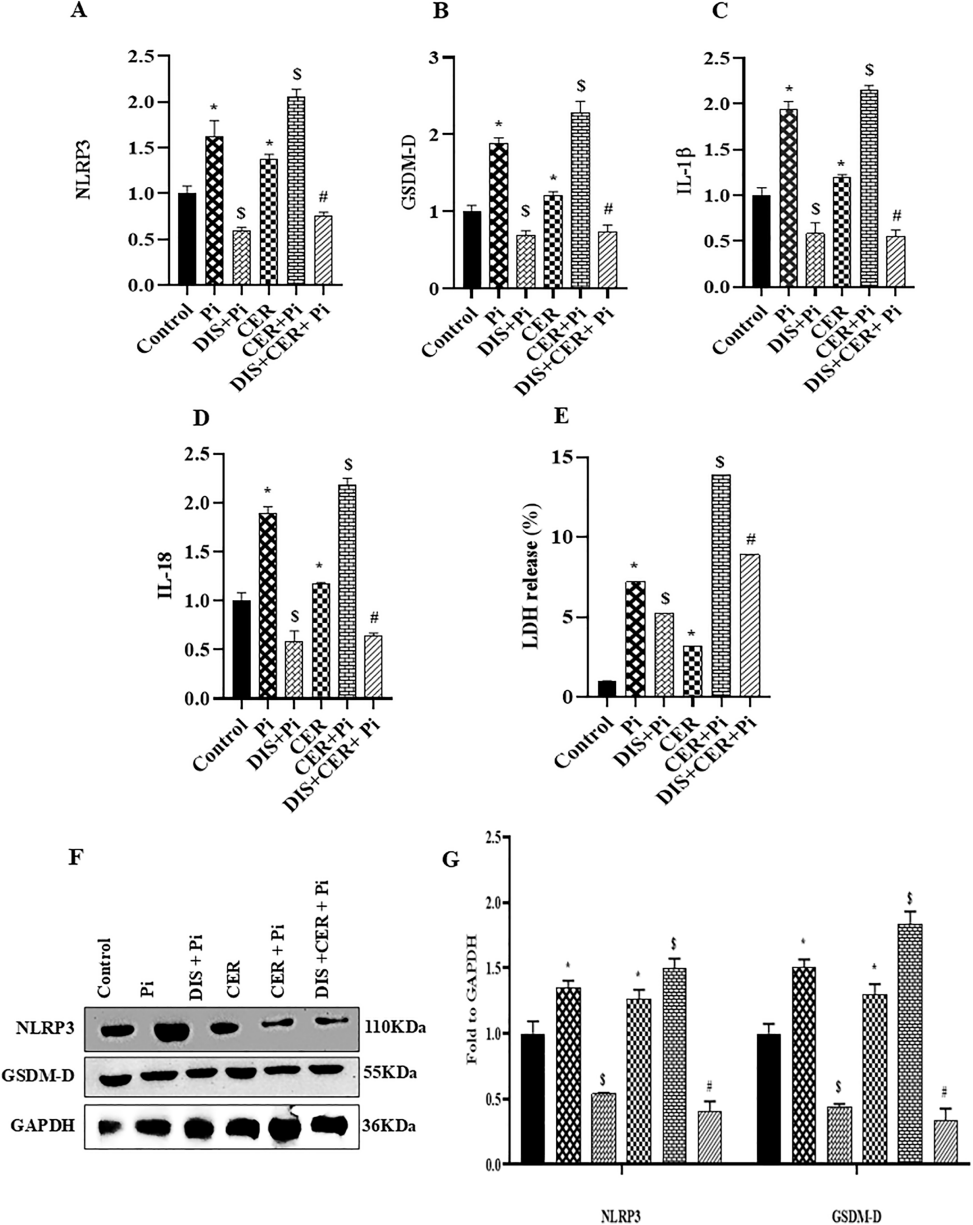
Effect of Gasdermin-D inhibition on CER-induced pyroptosis in P_i_*‐*treated VSMCs. Summarized bar graphs showed DIS significantly decreased mRNA expression of pyroptotic markers (A) NLRP3 (B) GSDM-D, proinflammatory cytokines (C) IL-1β (D) IL-18, and (E) Lactate dehydrogenase (LDH) release in CER treated P_i_‐induced VSMCs. (F-G) Representative western blots and summarized bar graph showed DIS significantly decreased protein expression of NLRP3 and GSDM-D in CER treated P_i_‐induced VSMCs. (n = 3–4). Results are presented as mean ±SD. Ceramide: CER, Disulfiram: DIS. *(P < 0.05) significant vs. control group; ^$^(P < 0.05) significant vs. P_i_ group; ^#^(P < 0.05) significant vs. CER + P_i_ group by One way ANOVA followed by Tukey’s test.

Furthermore, we explored the GSDM-D which is associated with the release of pro-inflammatory cytokines during pyroptosis, a prominent feature of pyroptotic cell death. Here, we observed that inhibition of GSDM-D by disulfiram, a pharmacological inhibitor of GSDM-D significantly decreased VC in P_i_‐induced and CER + P_i_ treated VSMCs as detected by Alizarin Red S staining in Fig 7(A), the summarized data is shown in the Fig 7(B). Also, mRNA expression of osteogenic marker such as RUNX2 was significantly downregulated whereas contractile marker SM22α was upregulated with disulfiram treatment in both P_i_ and CER + P_i_ treated groups as shown in Fig 7(C) and 7(D). This data was consistent with western blot results of phenotypic switch markers RUNX2 and SM22α in Fig 7(E) and 7(F). As shown in Fig 8(A)–8(D), prior disulfiram treatment caused significantly decreased mRNA expression of pyroptotic markers such as NLRP3, GSDM-D, IL-1β and IL-18 in P_i_ and CER + P_i_ treated VSMCs. As shown in Fig 8(E), prior disulfiram treatment showed significantly decreased LDH release in P_i_ and CER + P_i_ treated groups. Protein expression of pyroptotic markers GSDM-D and NLRP3 was significantly decreased with disulfiram in P_i_ and CER + P_i_ treated groups as shown in Fig 8(F) and 8(G). Hence, the data indicated that inhibition of GSDM-D, a prominent marker of pyroptotic pathway in VSMCs prevents their phenotypic transition and also blocked CER-induced pyroptosis during VC.

### Evogliptin alleviated ceramide-induced pyroptosis and phenotypic transition in P_i_*‐*treated VSMCs

Recently, it was reported that evogliptin ameliorates the mineralization of the primary human valvular interstitial cells (VIC), decreased expression of bone-associated and fibrosis-related genes thereby preventing valvular calcification during calcific aortic valve disease progression [26].

**Fig 9:**
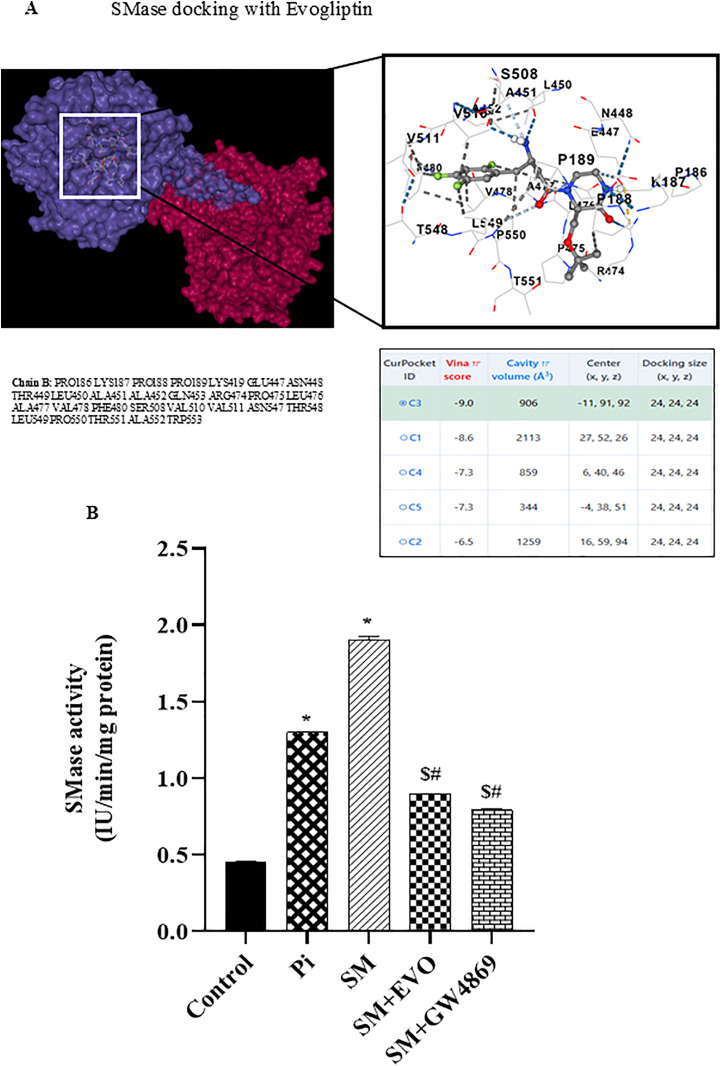
*In-silico* and *in-vitro* analysis of evogliptin with SMase. Molecular docking of evogliptin with SMase (A) 3D complex and amino acid binding of evogliptin to SMase. (B) Summarized bar graph of SMase activity assay. Sphingomyelinase: SMase, Sphingomyelin: SM. *(P < 0.05) significant vs. control group; ^$^(P < 0.05) significant vs. P_i_ group; ^#^(P < 0.05) significant vs. SM group by One way ANOVA followed by Tukey’s test.

Hence, we investigated whether evogliptin could modulate the CER-induced osteogenic changes and pyroptosis in P_i_ treated VSMCs. Firstly, we performed the molecular docking by *in-silico* analysis between the drug, evogliptin and target sphingomyelinase (SMase) enzyme (which hydrolyse sphingomyelin (SM) to CER in *de-novo* synthesis). The molecular docking analysis showed a close interaction between SMase and evogliptin as shown in Fig 9(A). The *in-silico* results indicated that evogliptin may lead to reduced SMase activity which in turn may prevent CER-induced cellular processes during VC. Under *in-vitro* conditions we observed that treatment with P_i_ and SM significantly increased SMase activity in SM group followed by P_i_ group while as prior treatment with evogliptin and GW4869, a specific inhibitor of SMase, significantly reduced SMase activity in SM-induced VSMCs (Fig 9B).

**Fig 10:**
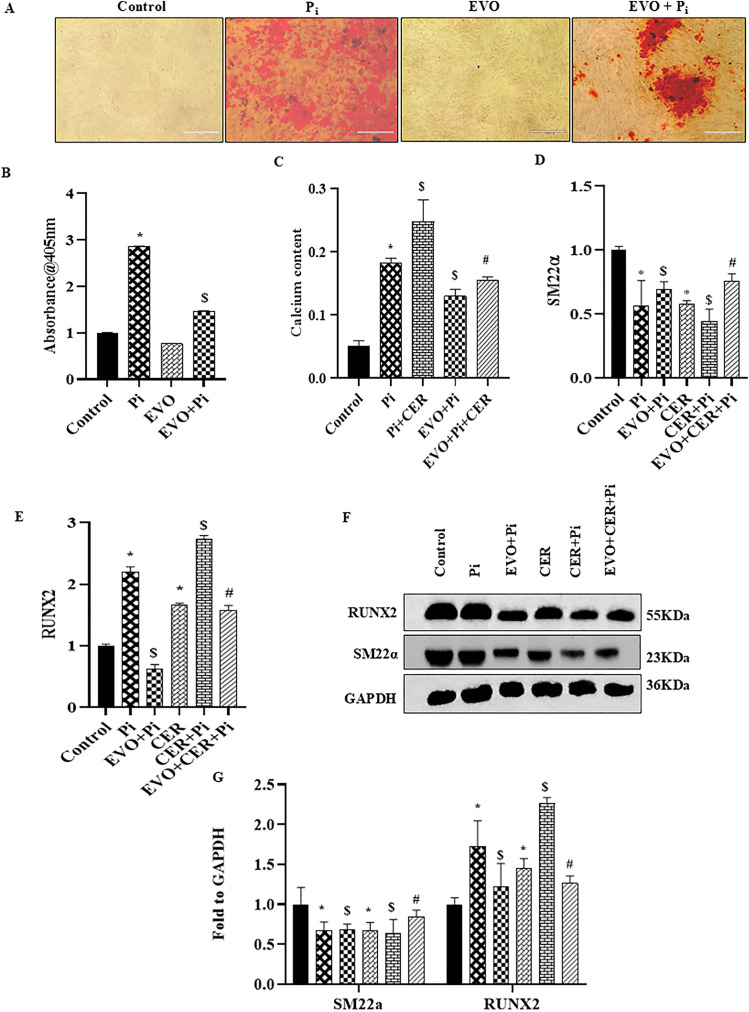
Evogliptin attenuates the phenotypic switch of VSMCs during CER treatment by decreasing the osteogenesis-associated genes *in-vitro.* (A) Representative images showed calcium deposition (red). (B, C) Summarized bar graph showed EVO significantly decreased calcium deposition and calcium content in CER treated P_i_-induced VSMCs. Bar graphs depicted EVO increased mRNA expression of (D) SM22α and decreased (E) RUNX2 in CER treated P_i_-induced VSMCs. (F-G) Representative western blots and summarized bar graph showed EVO significantly increased protein expression of SM22α and decreased RUNX2 in CER treated P_i_-induced VSMCs. (n = 3–4). Results are presented as mean ± SD. Ceramide: CER, Evogliptin: EVO. *(P < 0.05) significant vs. control group; ^$^(P < 0.05) significant vs. P_i_ group; ^#^(P < 0.05) significant vs. CER + P_i_ group by One way ANOVA followed by Tukey’s test.

**Fig 11:**
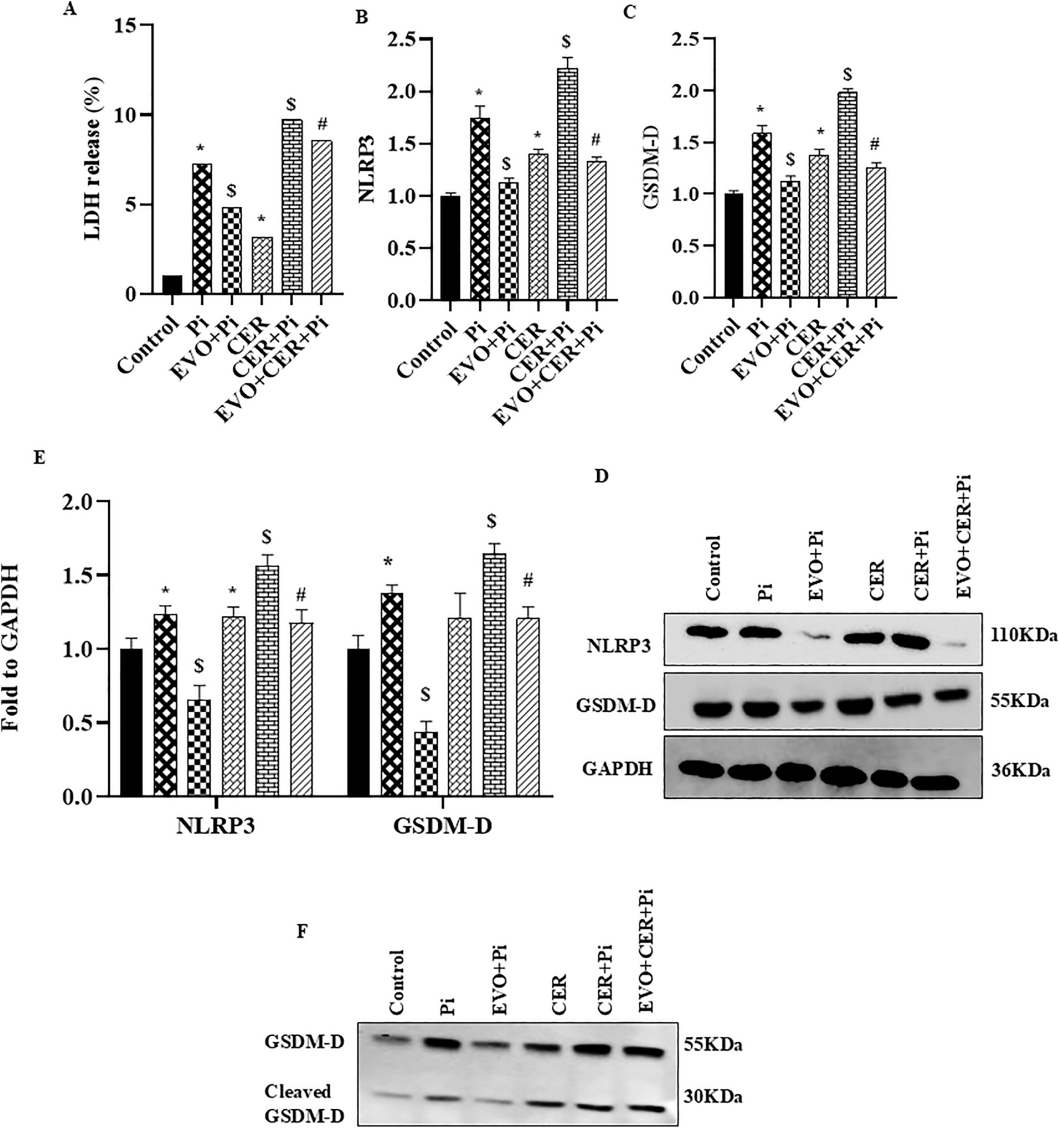
Evogliptin attenuates the pyroptotic cell death of VSMCs during CER treatment by decreasing the pyroptotic-associated genes and GSDM-D cleavage efficiency *in-vitro.* (A) Bar graph showed EVO significantly decreased Lactate dehydrogenase (LDH) release in CER treated P_i_‐induced VSMCs. Summarized bar graphs showed EVO significantly decreased mRNA expression of pyroptotic markers (B) NLRP3 and (C) GSDM-D in CER treated P_i_-induced VSMCs. (D-E) Representative western blots and summarized bar graph showed EVO significantly decreased protein expression of NLRP3 and GSDM-D in CER treated P_i_-induced VSMCs. (F) Representative Western blot showing cleavage and activation of GSDM-D into a full length GSDM-D and cleaved GSDM-D. (n = 3–4). Ceramide: CER, Evogliptin: EVO. *(P < 0.05) significant vs. control group; ^$^(P < 0.05) significant vs. P_i_ group; ^#^(P < 0.05) significant vs. CER + P_i_ group by One way ANOVA followed by Tukey’s test.

Literature cites, GW4869 reduced VC *in-vitro* [36]. Furthermore, we observed that evogliptin decreased calcium deposition as detected by Alizarin Red S staining (Fig 10A) and calcium assay during VC in P_i_ as well as in CER + P_i_ treated VSMCs, the summarized data is depicted in the Fig 10(B) and 10(C). Moreover, prior treatment of evogliptin attenuated the osteogenic conversion of VSMCs depicted by decreased mRNA expression of RUNX2 and augmented SM22α expression as shown in Fig 10(D) and 10(E). This data was consistent with western blot results of phenotypic switch markers SM22α and RUNX2 in Fig 10(F) and 10(G). In our earlier results, we observed that NLRP3 and GSDM-D contributes to CER-induced phenotypic transition and pyroptosis in VSMCs during VC. Here, we found that evogliptin significantly reduced LDH release (Fig 11A), mRNA and protein expression of pyroptotic markers such as NLRP3 and GSDM-D as shown in Fig 11(B)–11(E). GSDM-D is composed of an N-terminal region and a C-terminal region with inhibitory functions, on inflammasome activation, caspase-1 and/or caspase-11 cleave GSDM-D, releasing the N-terminal fragment that is the effector domain. The N-terminal domain is liberated which oligomerizes into the plasma membrane, resulting in pore formation. This pore formation results in loss of osmotic homeostasis, swelling of the cell, and then pyroptosis [37]. Next, we evaluated GSDM-D cleavage by western blot analysis and found that under different treatments there is cleavage and activation of GSDM-D into a full length GSDM-D showing bands at 55kDa and cleaved GSDM-D at 30kDa as shown in Fig 11(F). Also, the cleavage efficiency was high in P_i_ and CER + P_i_ groups which was reduced with prior treatment of evogliptin. Together, these results indicated that evogliptin alleviates VC by ameliorating SMase activity, blocking phenotypic transition and associated pyroptosis via modulation of NLRP3/GSDM-D mediated pathway in CER-induced VSMCs during VC.

## Discussion

In the present study, we investigated that evogliptin alleviates VC by ameliorating sphingomyelinase (SMase) activity, blocking phenotypic transition and associated pyroptosis via modulation of NLRP3/GSDM-D mediated pathway in CER-induced VSMCs during VC. Firstly, we observed that CER-enhanced calcification with augmented Ca/P deposition, and significantly increased expression of osteogenic markers such as RUNX2, OPN and BMP2 while as contractile markers such as SM22α and α-SMA were significantly decreased in P_i_-treated VSMCs. SMase catalyse hydrolysis of sphingomyelin (SM) to CER and phosphocholine, the studies showed that CER supplementation or acid sphingomyelinase (aSMase) deficiency reduced VC in VSMCs as well as in mouse models suggesting that acid SMase/CER is crucial upstream regulator of VC [38,39]. Recently, a study reported that ceramide synthase 5 (CerS5), a critical enzyme for C16 ceramide synthesis plays a crucial role in the valvular interstitial cells (VIC) and high‐fat/high‐cholesterol diet (HFHCD) fed mice during the development of aortic valve stenosis and inflammation [9]. Bhat *et al* demonstrated that lysosomal acid ceramidase regulates CER metabolism and plays a critical role in phenotypic transition, small extracellular vesicles (sEVs) secretion and lysosome-MVBs interaction in arterial SMCs, thereby plays a crucial role in arterial stiffening and calcification [10,40].

Literature reports a resilient relationship between pyroptosis and vascular disorders including pyroptosis of VSMCs [35,41] endothelial cells [42] involved in vascular remodeling. Since VSMCs are abundant cell type in vascular walls and play an essential role in maintaining vessel shape and function. Pyroptosis and NLRP3 inflammasome activation play a crucial role in phenotypic transition or transdifferientiation of VSMCs which result in cardiovascular disorders, including hypertension, atherosclerosis, VC and abdominal aortic aneurysm [43]. In the present study, we found significantly increased expression of pyroptotic markers such as NLRP3, GSDM-D, pro-inflammatory cytokines such as IL-1β and IL-18, increased LDH release upon P_i_ treatment as compared to control group. A study depicted that irisin suppresses VC via inducing autophagy and inhibiting VSMC pyroptosis in CKD-associated VC [17]. In β-glycerophosphate (β-GP)-stimulated VSMCs, augmented ROS, IL-1β, IL-18, LDH release, activated caspase3/GSDME and increased calcification was observed. However, ligustrazine or tetramethylpyrazine (TMP) inhibited caspase-3/GSDME mediated pyroptosis as well as pyroptotic markers and alleviated the progression of coronary artery calcification [44].

Since sphingolipids play a vital role in the pathology of cardiovascular diseases. Next, we explored the role of sphingolipid-CER-induced pyroptosis in VSMCs during VC. Literature cites, CER acts as a positive modulator of NLRP3 inflammasome formation and the release of inflammatory cytokine such as IL-1β in microglia cells [45]. During spinal cord injury, inhibition of ceramide synthase 5 expression in microglia significantly suppressed microglial pyroptosis mediated by NLRP3, thereby exerting neuroprotective effects [45]. In human umbilical vein endothelial cells (HUVECs), C8-Ceramide aggravates vascular endothelial cell damage which stimulated reactive oxygen species (ROS) generation and also increased cell permeability by inducing cell pyroptosis [46]. HFHCD fed mice exhibited increased protein levels of sphingomyelin synthase 1, an enzyme that generates SM and diacylglycerol (DAG) from *de novo*-synthesised CER. In the supernatant of isolated HFHCD hepatocytes, enhanced release of LDH and increased levels of pyroptotic marker GSDMD-N was observed, a key player in pyroptotic cell death. Besides, conditioned media from these pyroptotic HFHCD hepatocytes activated the NLRP3 in Kupffer cells indicating that NLRP3 activation is crucial player in pyroptosis [47]. A study reported that nerve injury-induced protein 2 (NINJ2) deficiency, a cell adhesion molecule, resulted in alteration of lipids including CER which was increased in NINJ2-KO MCF7 cells. Furthermore, the study showed that in bone marrow derived macrophages isolated from NINJ2-KO mice, NINJ2-KO MCF7 and Molt4 cells, there was significantly increased LDH release and NLRP3 expression which promotes plasma membrane rupture and pyroptosis [48]. Orosomucoid-like protein 3 (Ormdl3) gene which is associated with asthma have been found to alter sphingolipid levels including CER, and augmented the expression of pyroptotic markers such as NLRP3 and GSDM-D in lung tissues of obese asthmatic mice and human bronchial epithelial cells [49–51]. Together, these studies indicated that sphingolipids including CER ameliorate pyroptosis via NLRP3 and GSDMD. Our findings are in tune with the above studies where we observed that CER treatment in P_i_‐induced VSMCs significantly augmented expression of pyroptotic markers such as NLRP3, GSDM-D, IL-1β, IL-18 and augmented LDH release during calcification. Also, our SEM results showed that prior treatment with CER enhanced pyroptotic features of VSMCs including cell membrane rupture and blebbing during VC. Moreover, our results regarding phenotypic switch of VSMCs were in line with literature which depicted increased osteogenic trans-differentiation of VSMCs upon CER treatment. Several other *in vitro* and *in vivo* studies revealed that NLRP3 and pyroptosis aided phenotypic transition and vascular modelling that contributes to the pathogenesis of VC [52]. Our findings provide strong and direct evidence that sphingolipid-CER is associated with pyroptosis in VSMCs during VC.

Various studies reported that NLRP3 plays a crucial role in atherosclerosis, vascular remodelling and facilitates VSMC pyroptosis through autophagy induction and downregulation of ROS generation [13,17,53]. In CKD, trimethylamine N-oxide (TMAO), a gut microbiota-dependent product, induced NLRP3 inflammasome activation and NF-κB signals which promotes calcification in VSMCs [11]. In calcified VSMCs, disorganisation of α-SMA was associated with pyroptotic features of these cells including maturation of caspase-1, increased LDH release and cleavage of GSDM-D [43]. In uremic end-stage kidney disease patients with moderate/severe calcification, augmented levels of serum GSDM-D were found, and there was positive correlation between GSDM-D and caspase-1, IL-1β, IL-6 [54]. Recently, in HUVECs, CER caused vascular permeability, also increased expression of NLRP3, GSDMD, IL-1β, IL-18 and LDH release via ROS-dependent thioredoxin interacting protein (TXNIP) pathway leading to pyroptosis in HUVEC [46].We also observed pharmacological inhibition by MCC950 (a specific NLRP3 inhibitor) and disulfiram (a GSDM-D inhibitor) in VSMCs blocked CER-induced phenotypic transition and pyroptosis during VC. MCC950 and disulfiram significantly decreased expression of phenotypic switch marker such as RUNX2 as well as pyroptotic markers such as NLRP3, GSDM-D, IL-1β, IL-18, and LDH release whereas contractile marker SM22α was increased with or without CER treatment in P_i_*‐*induced VSMCs. From this data we inferred that NLRP3/GSDM-D signaling pathway contributes to CER-induced pyroptosis and phenotypic switch in VSMCs during VC.

Evogliptin, a DPP-4 inhibitor and anti-diabetic drug had positive impact on incidences related to various cardiovascular events, and cardiac tissue distribution of evogliptin was found to be highest [28,55]. Recently, a clinical trial of evogliptin treatment showed approximately 25% annual relative reduction in aortic valve calcification progression [56]. In healthy subjects, evogliptin administration showed good tolerability in a dose of 1.25−60 mg with no subjects showed signs of hypoglycaemia. However, to halt non-glycaemic function more clinical efficacy need to be fully elucidated [26]. As mentioned, evogliptin is an antidiabetic drug, recently few studies reported its cardioprotective effects. Here, the present study added atheroprotective evidences to the existing literature particularly effect of evogliptin on sphingolipid such as CER-induced phenotypic transition and pyroptosis in VSMCs which contributes to the underlying pathology of arterial calcification and atherosclerosis. Studies in human VIC and animal models observed that evogliptin decreased calcification, fibrosis and cytokine release via modulating NFκB/TNFα/nitric oxide synthase pathway and Insulin like Growth Factor-1(IGF-1) signaling [26,27]. In liver cells and animal models, evogliptin reduced liver inflammation and fibrosis through autophagy via NRF2/HO1 signaling [25]. Recently, in 2023, it was reported that evogliptin reduced lipotoxicity, mitochondrial damage and fibrosis in diabetic animal models via modulating PPARγ/CD36 pathway [57]. However, its role in the inflammatory cell death, pyroptosis, in vascular cells has not been explored yet.

**Fig 12:**
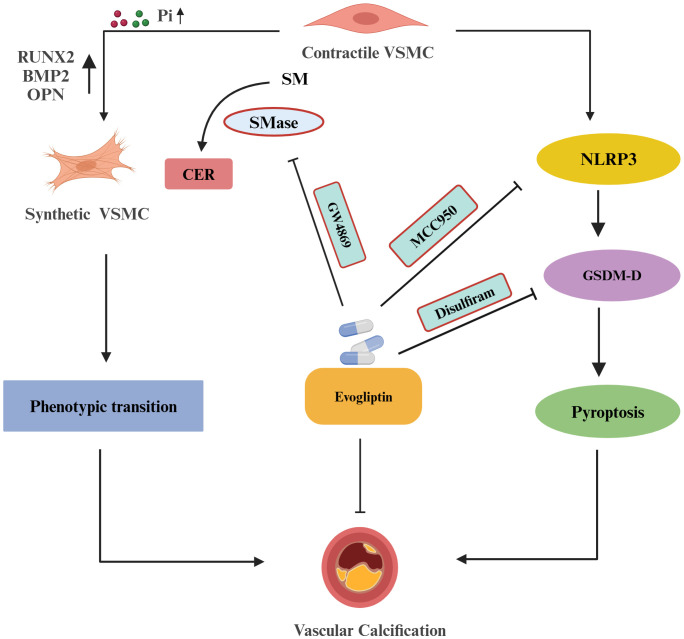
Schematic model indicates that CER enhanced the phenotypic transition and pyroptosis in VSMCs, and evogliptin alleviated CER-induced pyroptosis associated with decreased SMase activity via NLRP3/GSDM-D mediated pathway in VSMCs.

Firstly, to evaluate the effect of evogliptin on sphingolipid pathway in VC, we investigated its impact on SMase activity in P_i_ and GW4869 (specific pharmacological inhibitor of SMase) treated VSMCs. We performed the molecular docking by *in-silico* analysis between the drug, evogliptin and target sphingomyelinase (SMase) enzyme which hydrolyse SM to CER in *de-novo* synthesis. Interestingly, molecular docking analysis showed a close interaction between SMase and evogliptin. Moreover, our *in-silico* data was supported by *in-vitro* findings which depicted that evogliptin significantly decreased SMase activity, the decrease was at par with GW4869. Next, we observed evogliptin significantly decreased LDH release associated with reduced expression of other pyroptotic markers such as NLRP3 and GSDM-D and pro-inflammatory cytokines such as IL-1β and IL-18 during calcification. Also, we evaluated GSDM-D cleavage, a well-known mechanistic signaling pathway for pyroptosis by western blot analysis, and found that under different treatments there is cleavage and activation of GSDM-D into a full length GSDM-D showing bands at 55kDa and cleaved GSDM-D at 30kDa. The cleavage efficiency was high in phosphate induced VSMCs which was further enhanced with CER treatment and was reduced with prior treatment of evogliptin. We also found sphingolipid such as CER aided the pyroptotic process during VC as observed in earlier results which was alleviated with prior treatment of evogliptin. In Apo E-/- mice fed on high-fat diet and endothelial cells *in-vitro*, evogliptin reduced atherosclerotic plaque, macrophage infiltration into lesion and improve plaque stability via increasing collagen to necrotic core ratio, also reduced vascular inflammation in ECs via suppressing NF-κB activation and adhesion molecules which suggest that evogliptin has potential to target arterial inflammation [26,58]. The present study provided evidences regarding effect of evogliptin in the osteogenic conversion of VSMCs and sphingolipid pathway which together contributes to vascular calcification. This may be the first report which demonstrates that sphingolipid such as CER contributes to pyroptosis in VSMCs during calcification which was alleviated by evogliptin. All-together, these findings indicate that sphingolipid-ceramide enhanced the mineralization, calcification and pyroptosis in P_i_‐induced VSMCs during VC. Inhibition of NLRP3 inflammasome and GSDM-D, a prominent marker of pyroptotic pathway blocked phenotypic transition and CER-induced pyroptosis during VC (Fig 12). Furthermore, we found that evogliptin alleviates VC by ameliorating SMase activity, blocking phenotypic transition and associated pyroptosis via modulation of NLRP3/GSDM-D mediated pathway.

The limitation of the present study is *in-vivo* validation of these findings and exploring other mechanistic pathways contributing cardiovascular disorders such as atherosclerosis and vascular calcification. Also, exploring more biochemical assays including lipidomics to support metabolic changes in pyroptosis of VSMCs during vascular calcification. Furthermore, experiments such as atomic force mass spectroscopy (AFM) are required to examine whether evogliptin reduces the vascular stiffness and calcification that are associated with diabetes.

In future studies, we will explore whether evogliptin ameliorate the collagen-integrin-β1/FAK signaling and modulate cytoskeletal remodeling which are associated with arterial stiffness, underlying pathology of cardiovascular diseases including diabetes.

## Conclusion

In summary, our study demonstrated that evogliptin alleviates CER-induced pyroptosis associated with decreased SMase activity via NLRP3/GSDM-D mediated pathway in VSMCs during calcification. Our findings are consistent with the literature which recently reported the beneficial and pleiotropic effects of evogliptin on cardiovascular disorders, and added to insights that encourage the use of evogliptin to prevent the development of calcification and pyroptosis in VSMCs which contributes to the underlying pathology of cardiovascular diseases.

## Supporting information

S1 Raw Images Raw data. (DOCX)

S1 Data Minimal data sets. (ZIP)
