# Supplementary material for: Evogliptin prevents ceramide-induced pyroptosis during calcification via modulation of NLRP3/GSDM-D mediated pathway in Vascular Smooth Muscle Cells
Source: PLoS One. 2025 Dec 3;20(12):e0337200. doi: 10.1371/journal.pone.0337200 (PMC12674538; doi:10.1371/journal.pone.0337200)
Supplement: S1 Raw Images — (DOCX) [file pone.0337200.s001.docx]

**Raw data of Western Blotting**

**Figure 1**

**C P_i_**

**
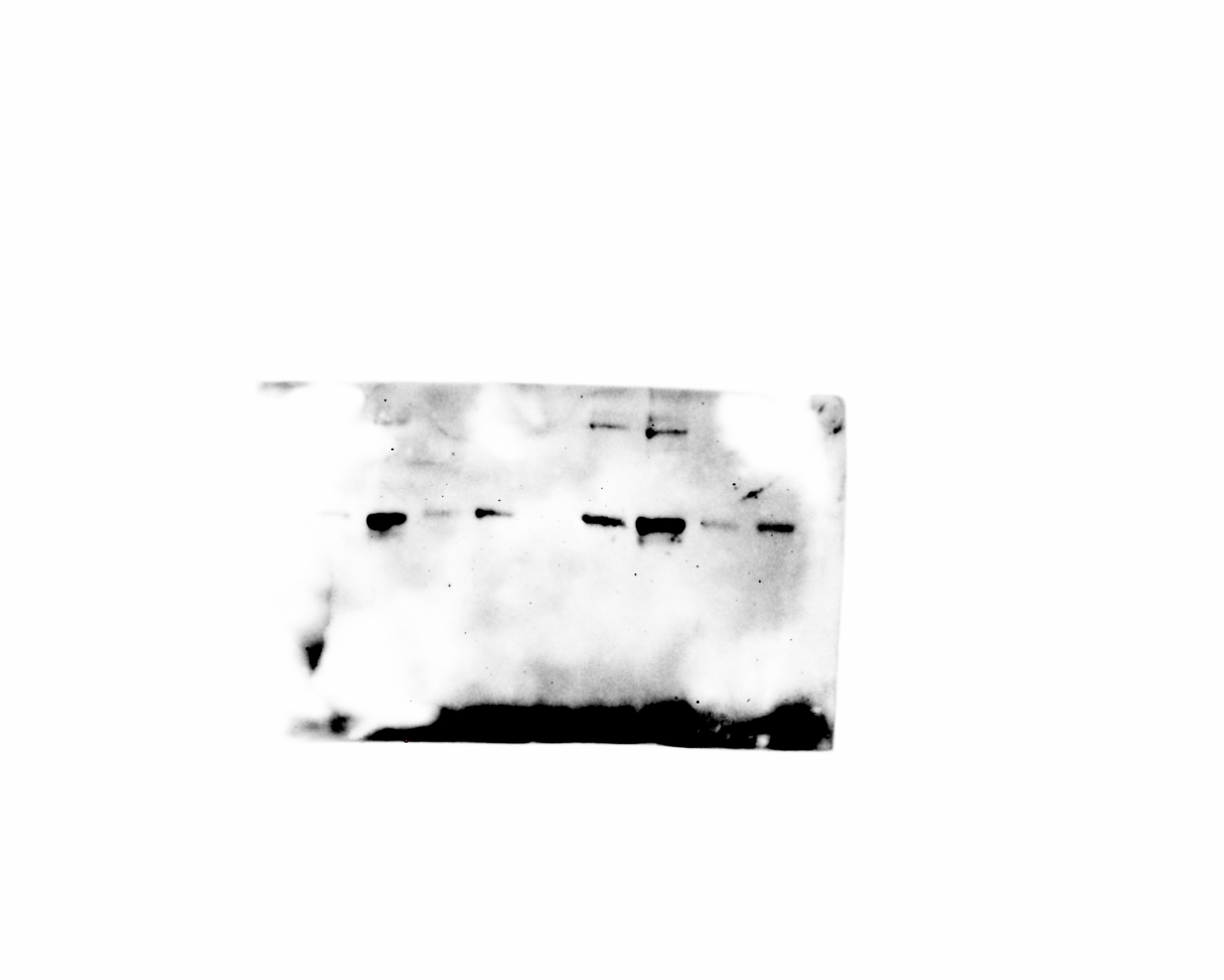
**

**OPN**

**
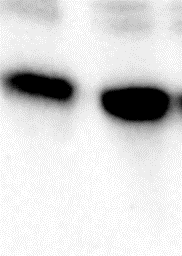
**

**RUNX2**

**
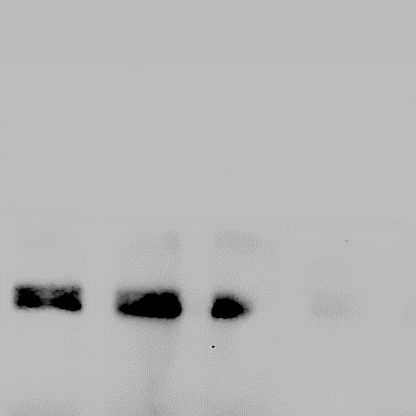
**

**SM22α**

**
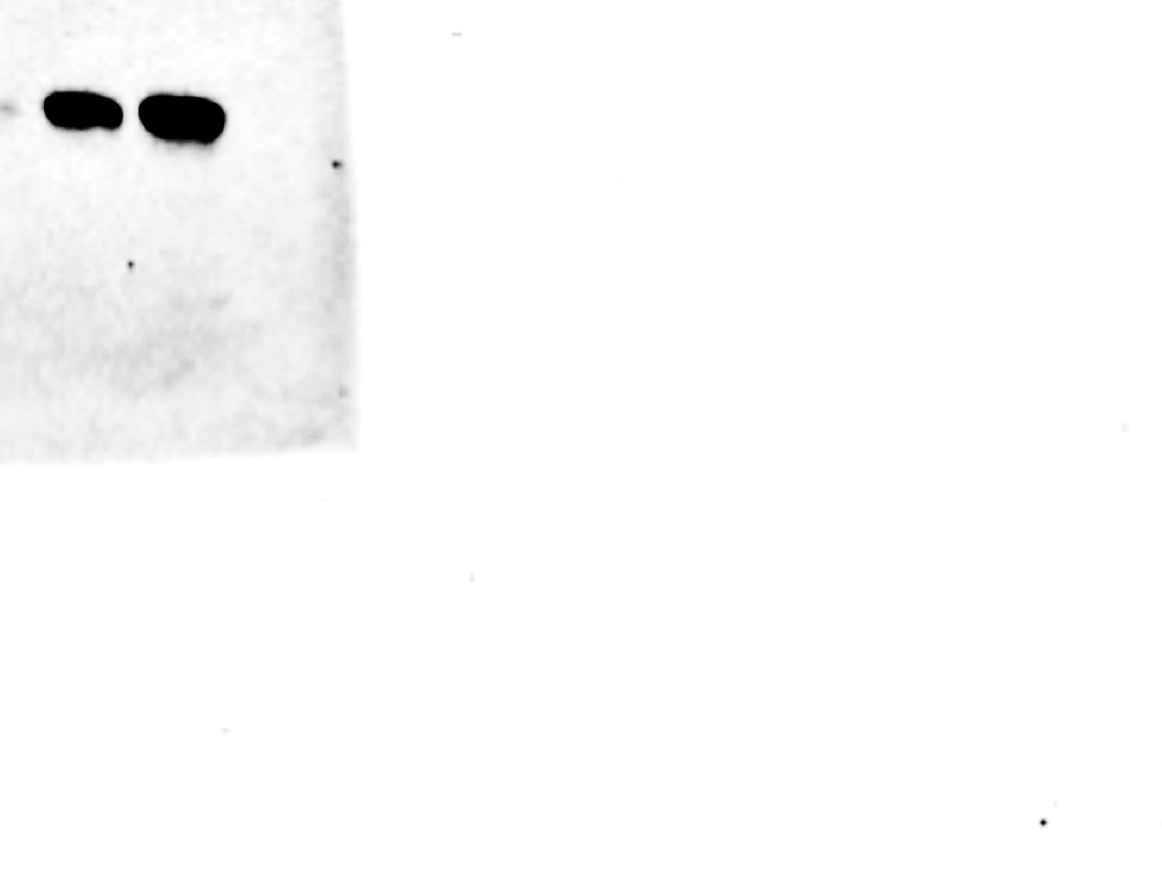
**

**GAPDH**

Control: C

Phosphate: P_i_

**Figure 2**

**C P_i_  CER CER +P_i_**


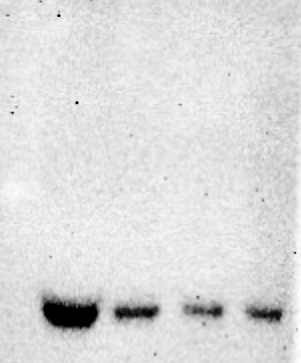


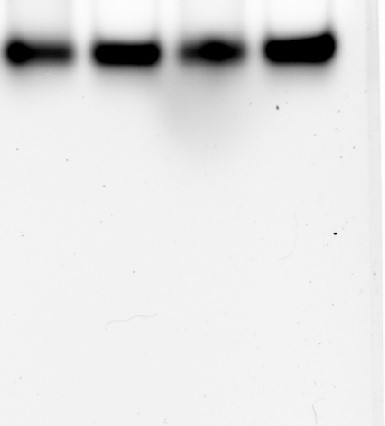

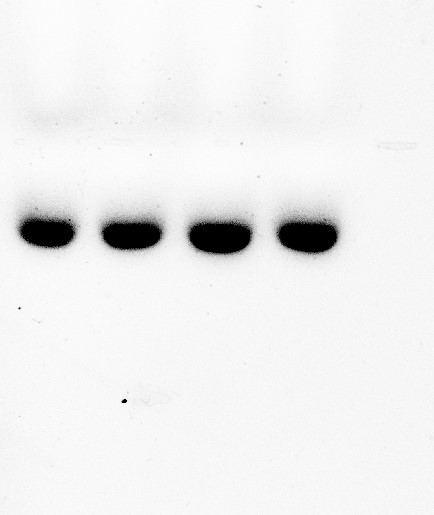


Control: C

Phosphate: P_i_

Ceramide: CER

GAPDH

RUNX2

SM22α

**C P_i_  CER CER +P_i_**

**C P_i_  CER CER +P_i_**


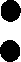

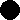


**Figure 4**

**C P_i_  CER CER +P_i_**

**
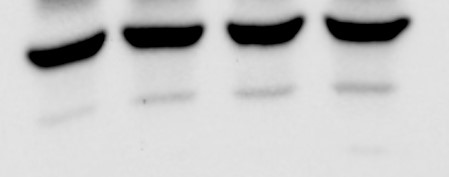

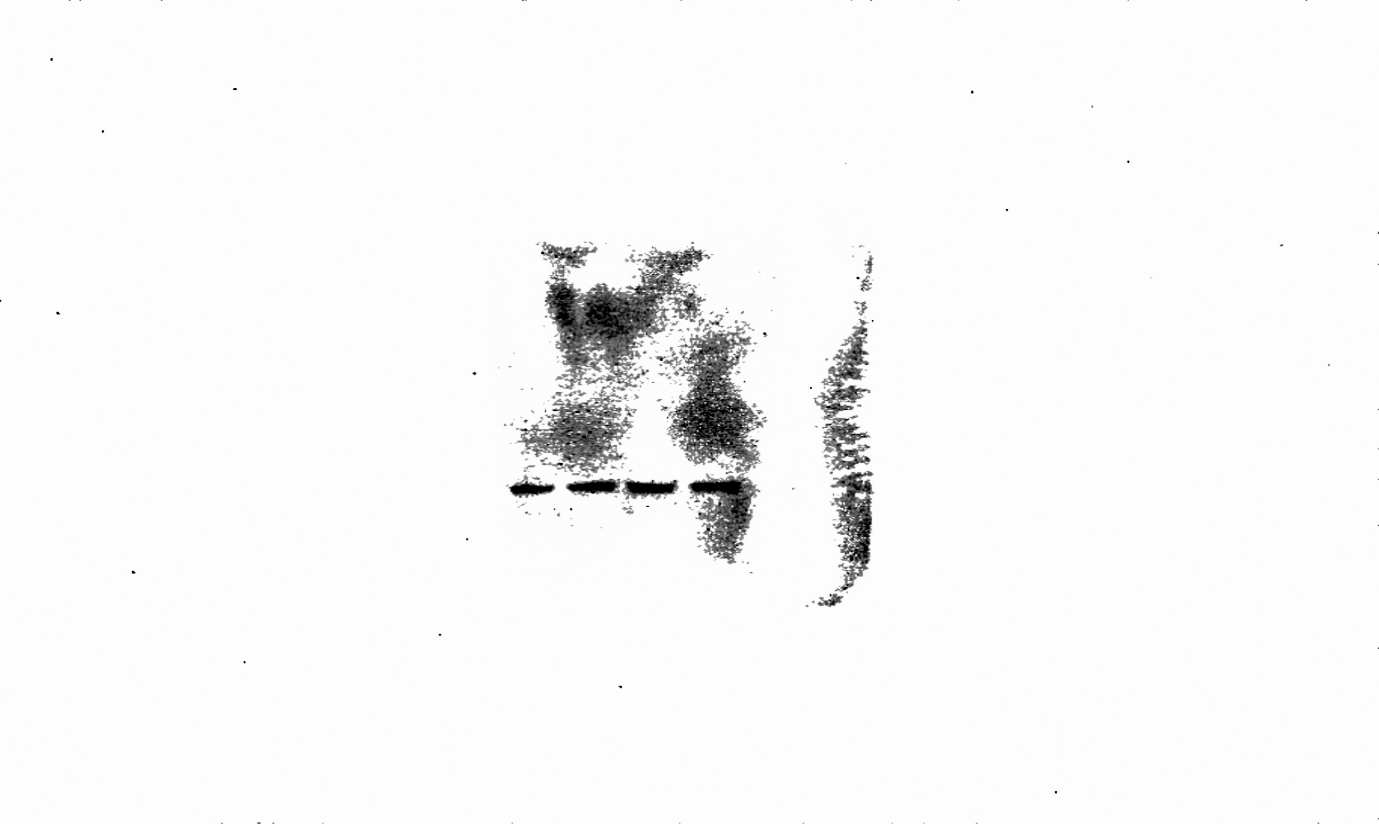

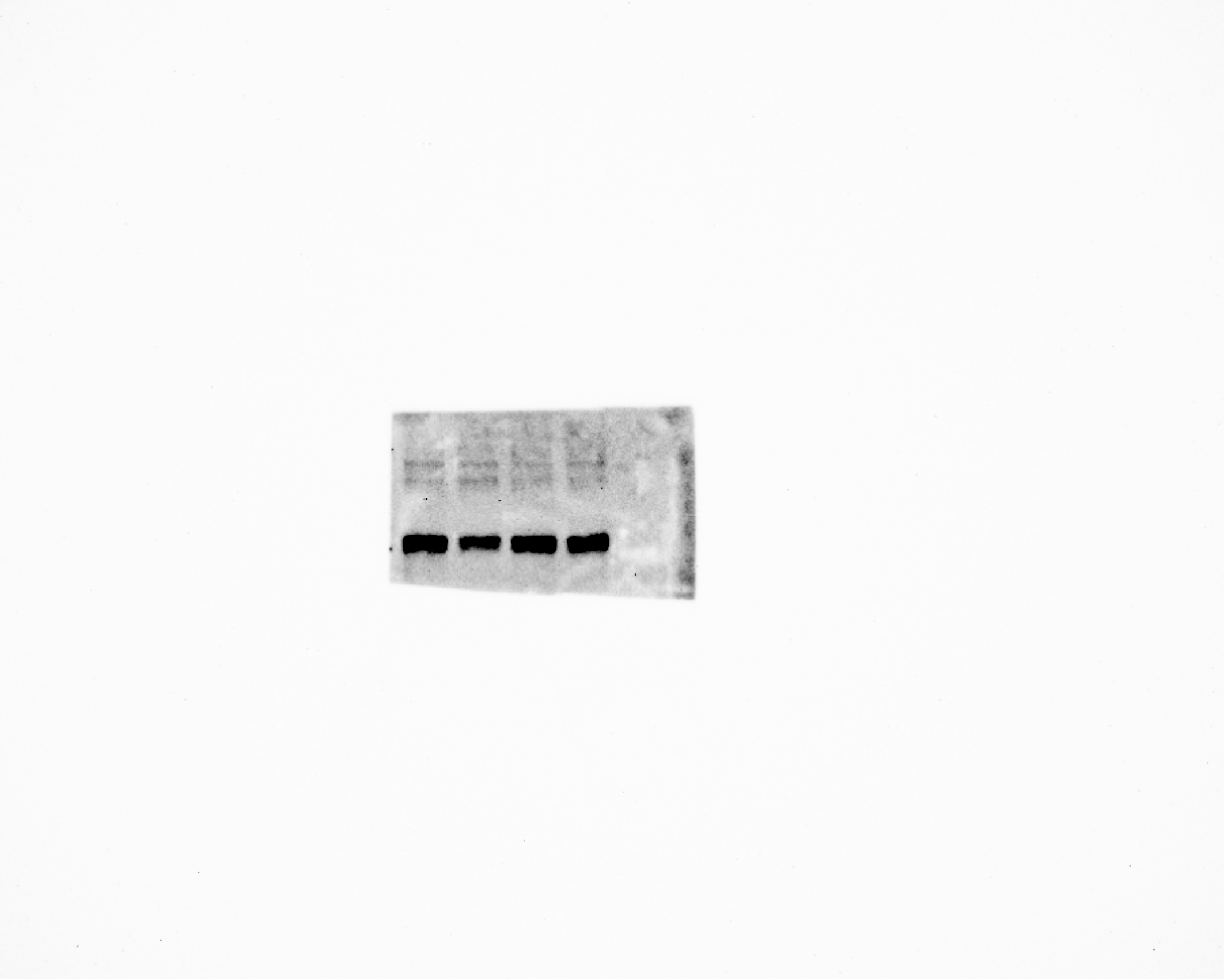
**

Control: C

Phosphate: P_i_

Ceramide: CER

GAPDH

**C P_i_  CER CER +P_i_**

GSDM-D

NLRP3

**C P_i_  CER CER +P_i_**

**Figure 5**

**C P_i_  M+P_i_ CER CER +P_i_ M+CER+P_i_**

**
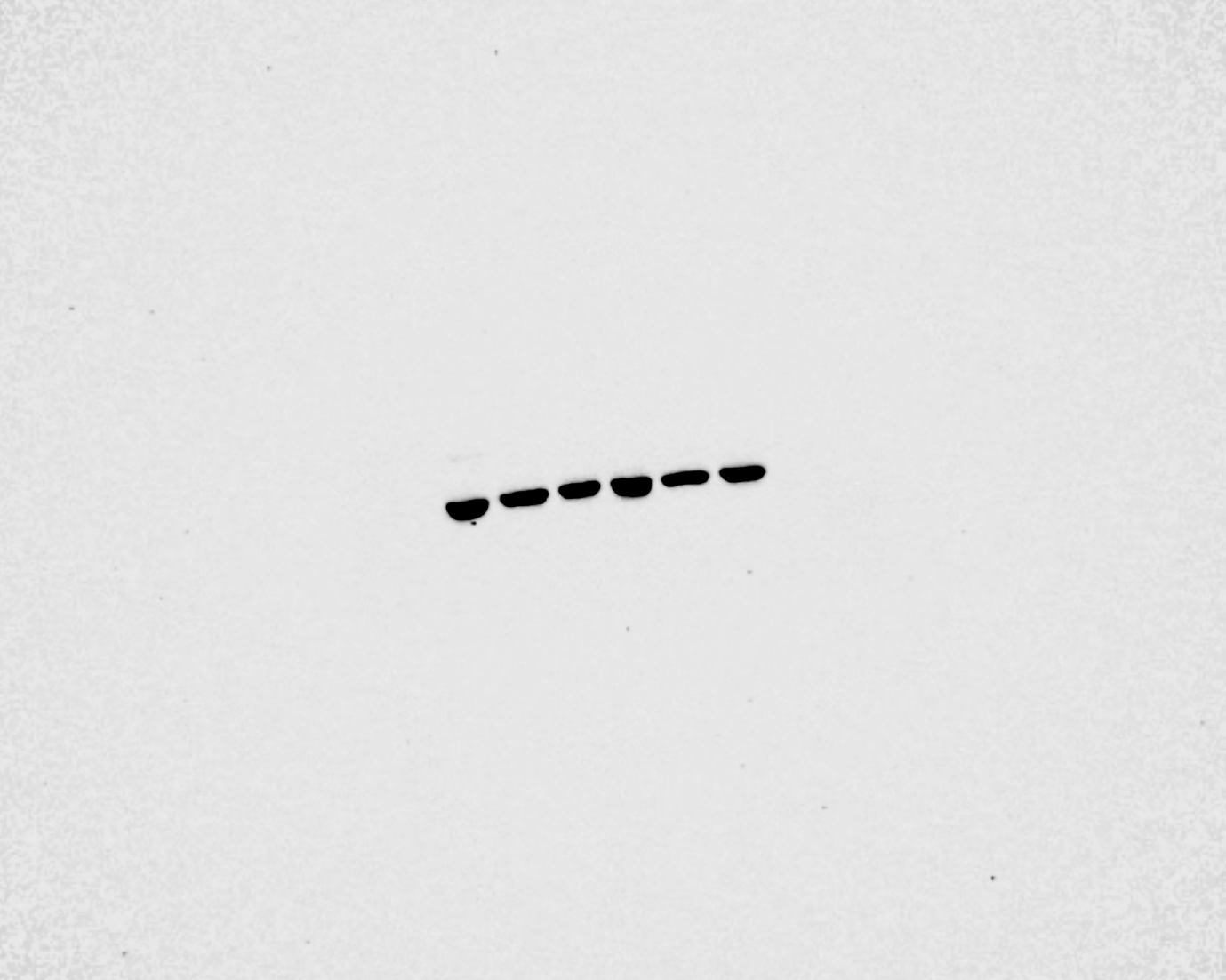
**

RUNX2

**
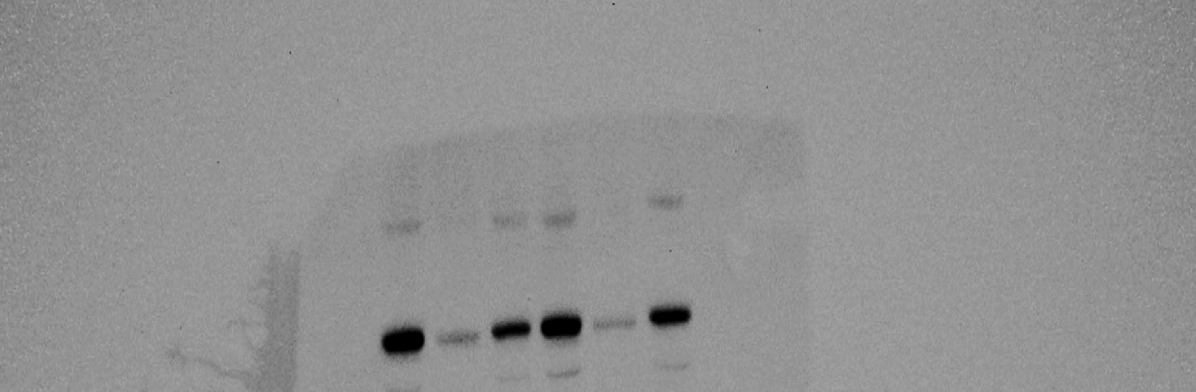
**

SM22α

**
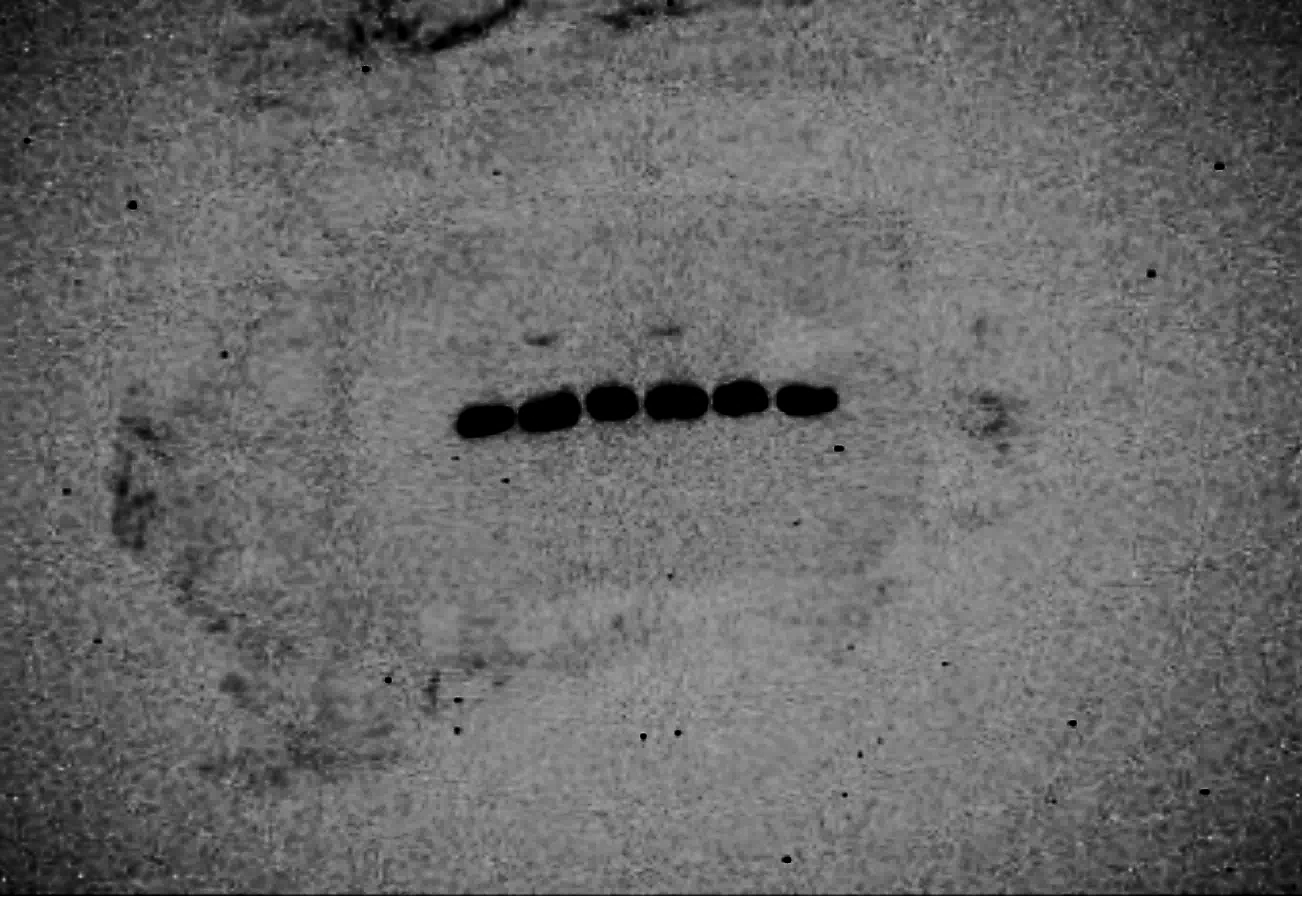
**

GAPDH

Control: C

Phosphate: P_i_

Ceramide: CER

MCC950: M

**Figure 6**


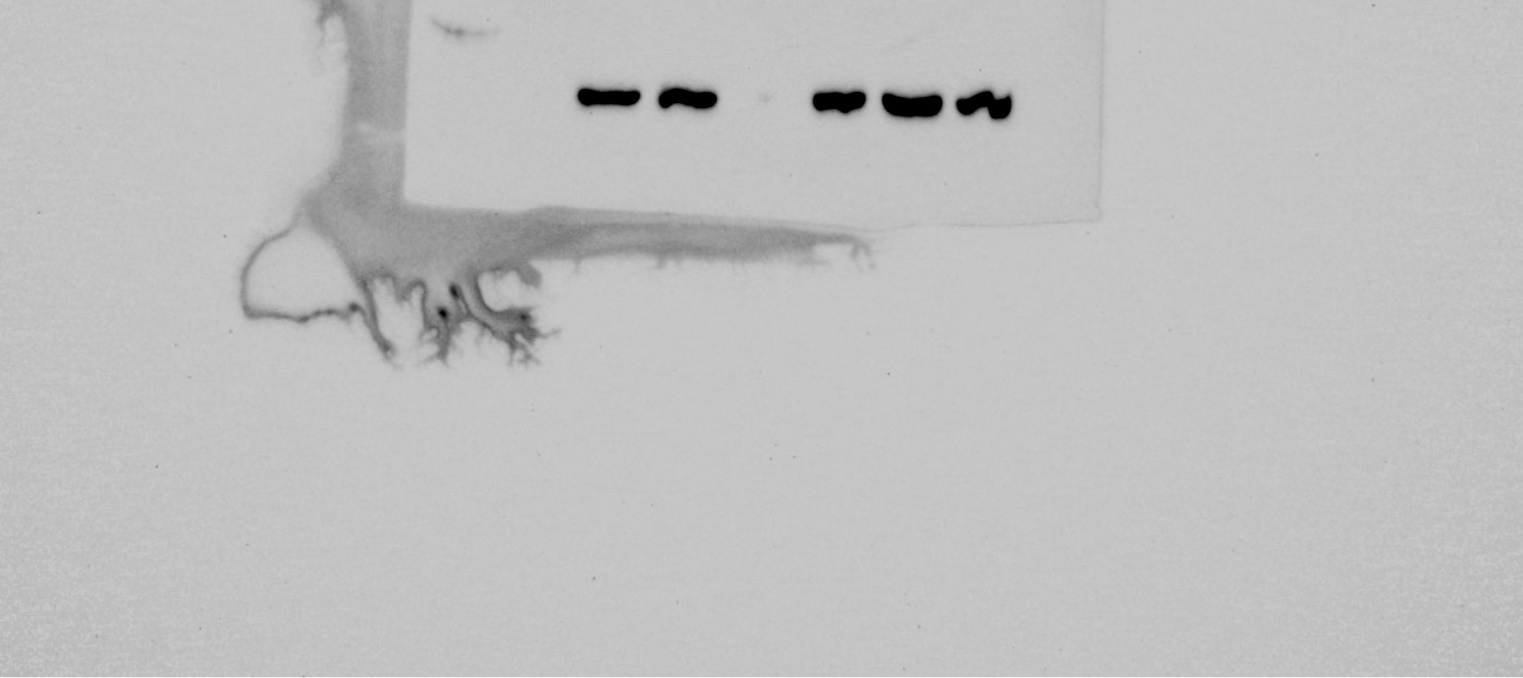

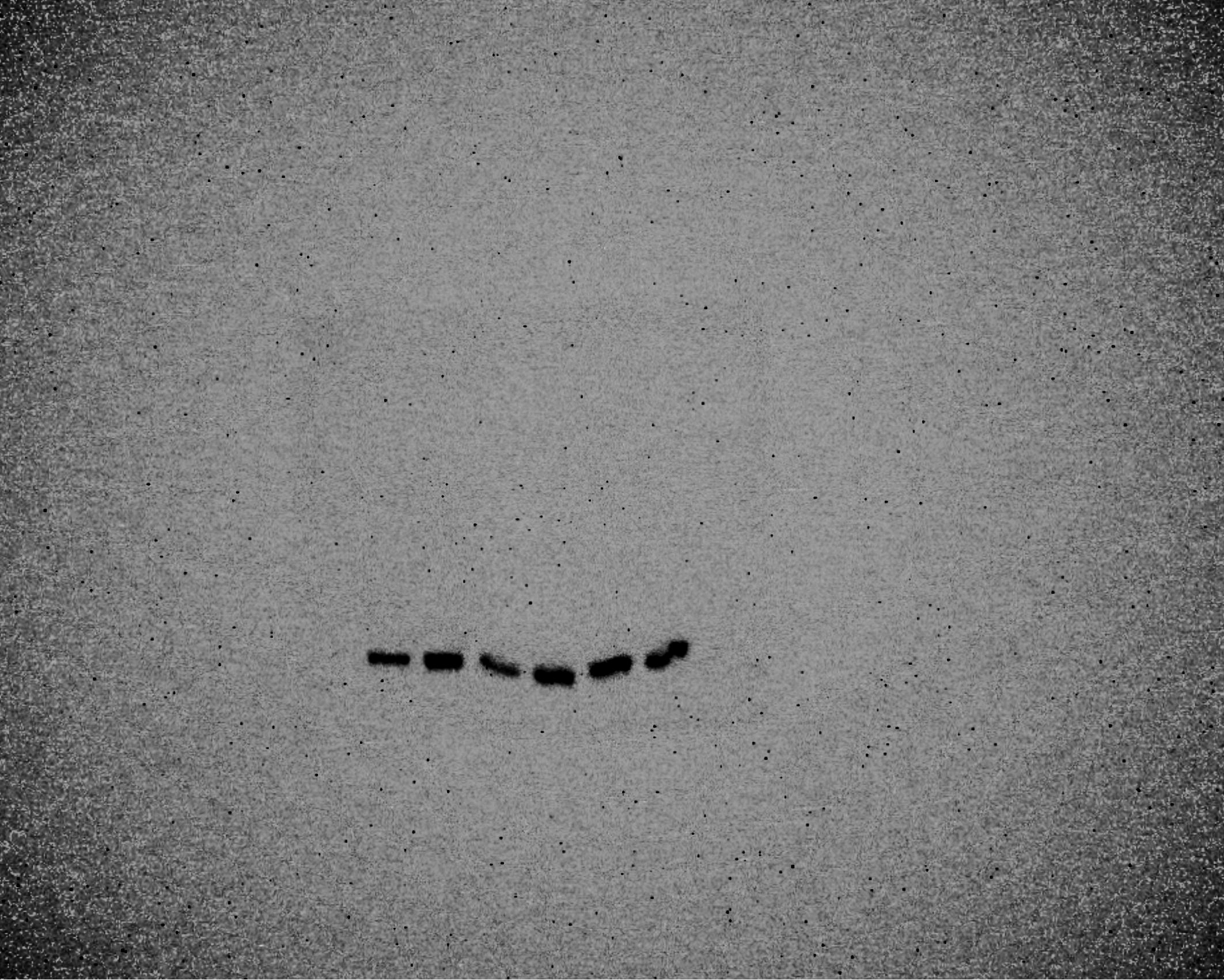

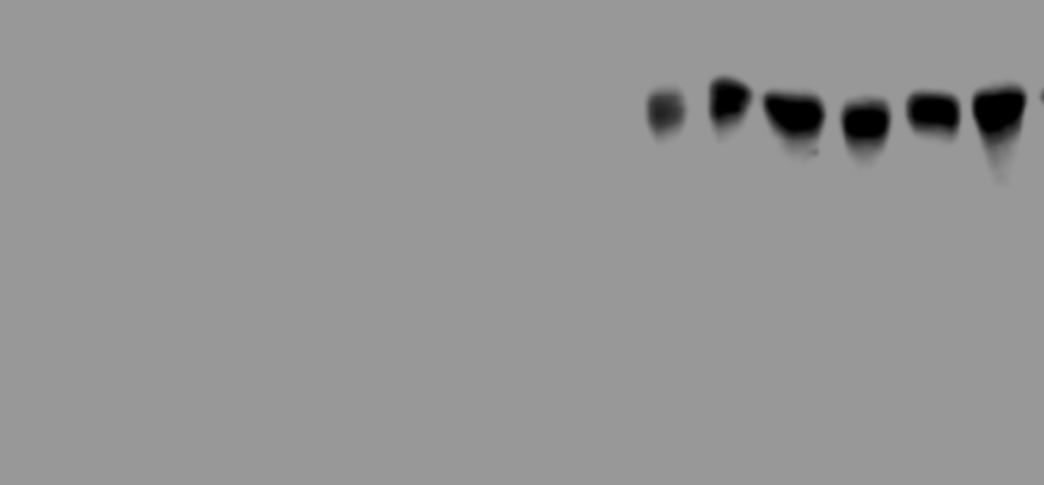


Control: C

Phosphate: P_i_

Ceramide: CER

MCC950: M

NLRP3

GSDM-D

GAPDH

**C P_i_  M+P_i_ CER CER +P_i_ M+CER+P_i_**

**Figure 7**

**C P_i_  D+P_i_ CER CER +P_i_ D+CER+P_i_**


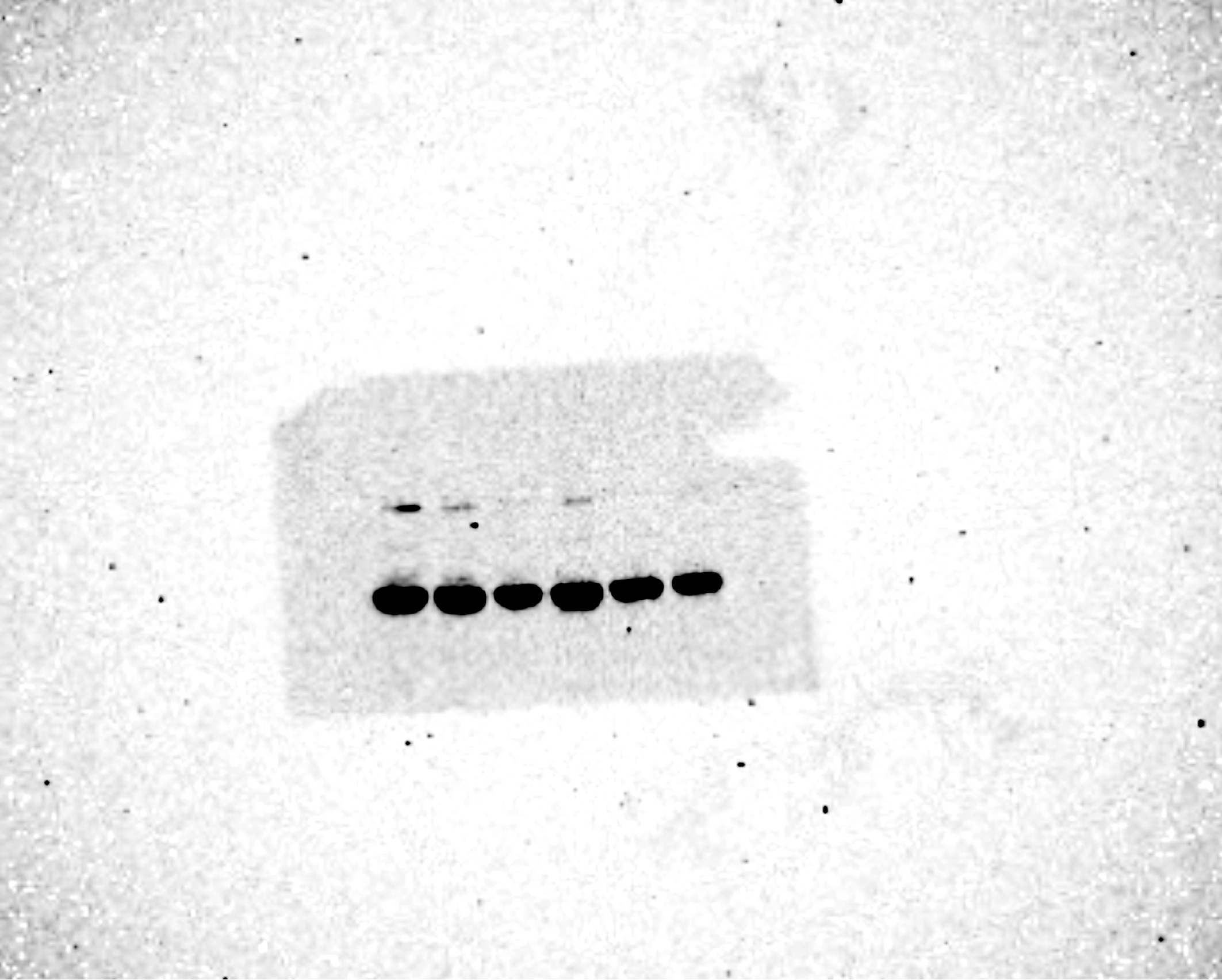


GAPDH


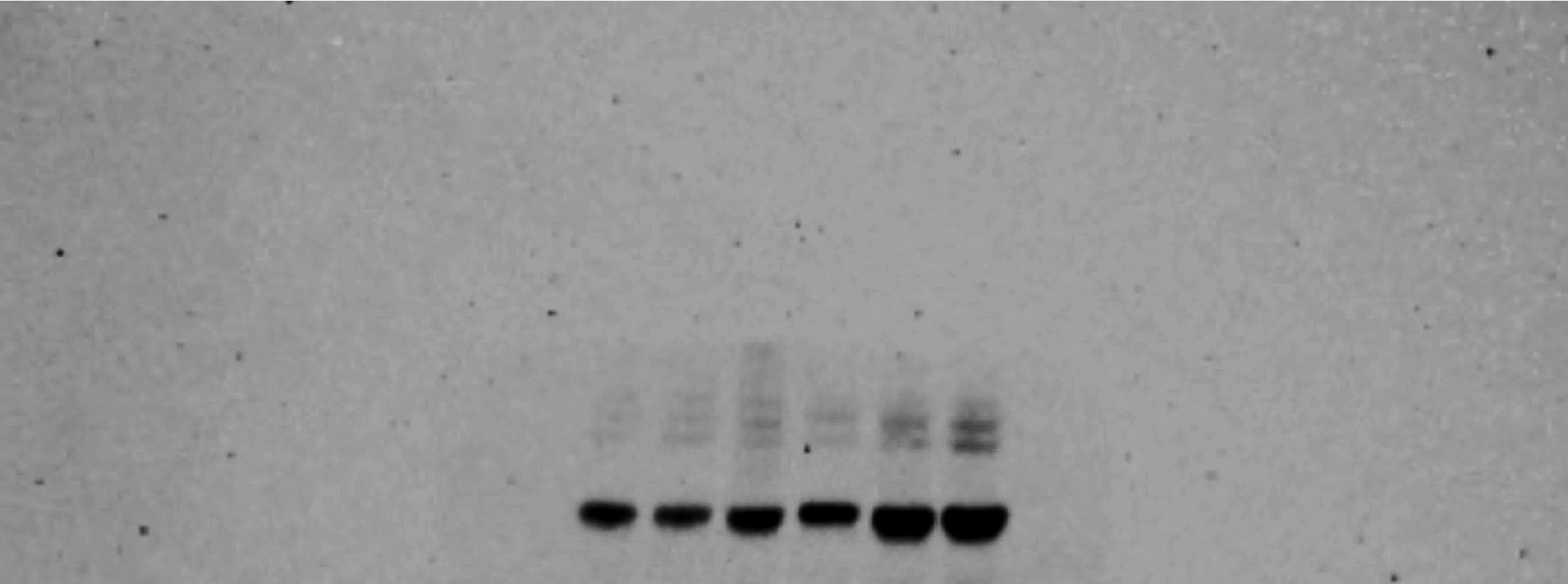

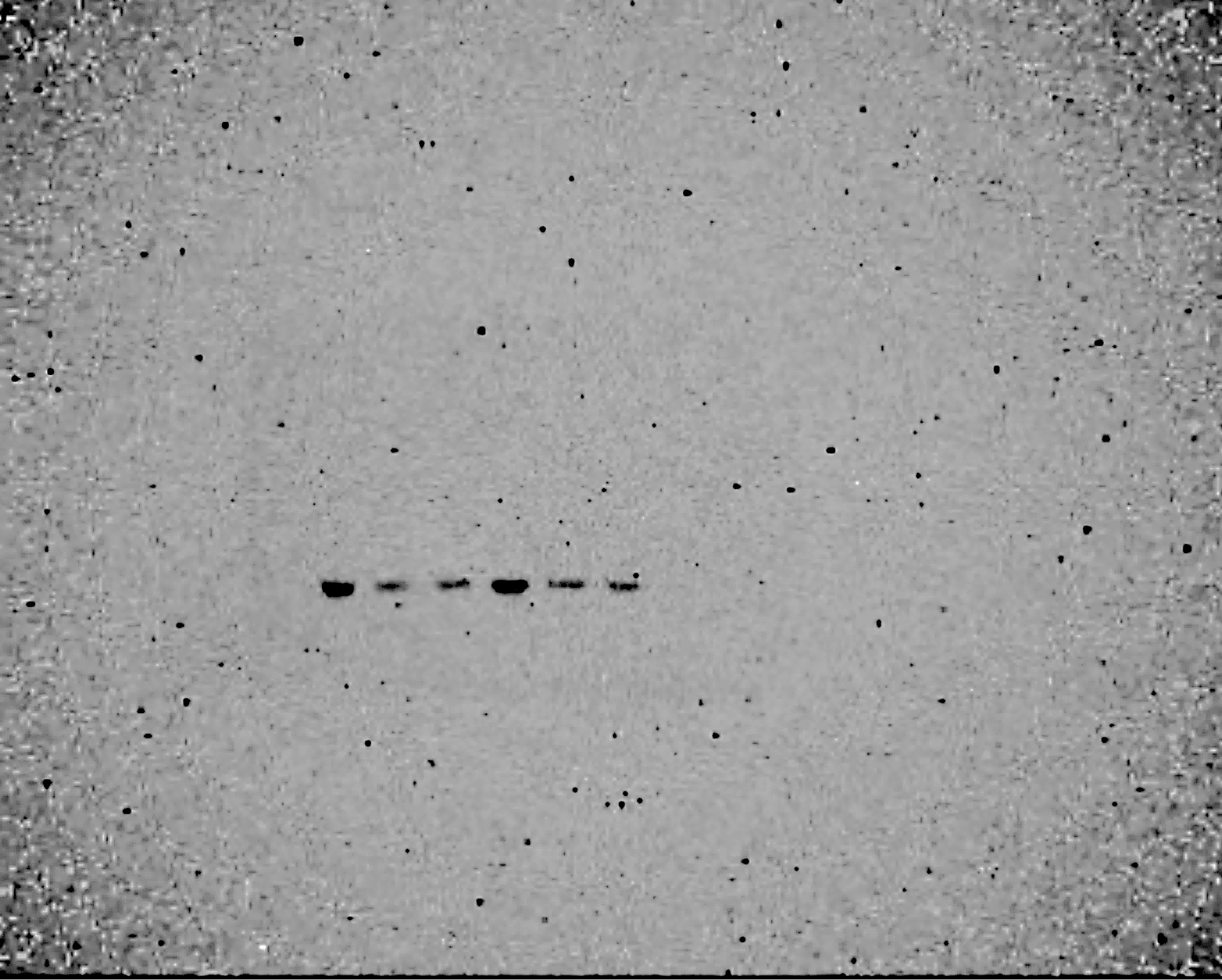


Control: C

Phosphate: P_i_

Ceramide: CER

Disulfiram: D

RUNX2

SM22α

**Figure 8**

**C P_i_  D+P_i_ CER CER +P_i_ D+CER+P_i_**

**
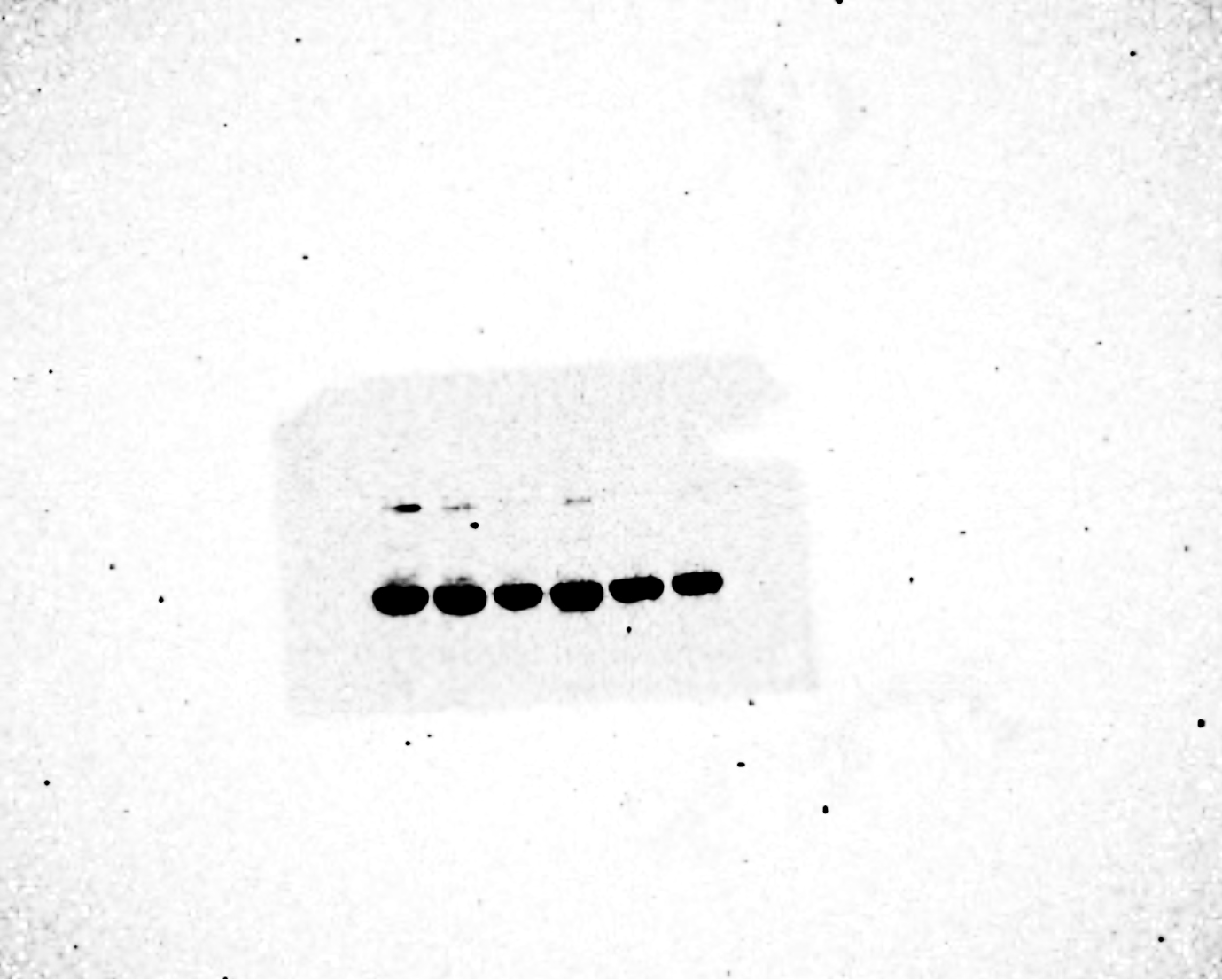
**

GAPDH


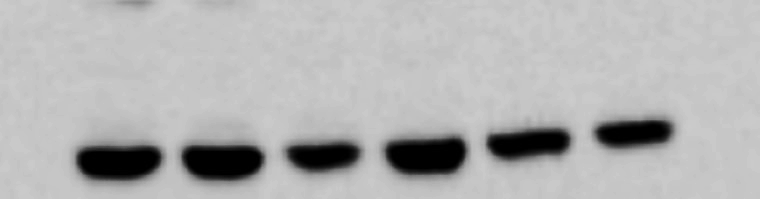


GSDM-D

**
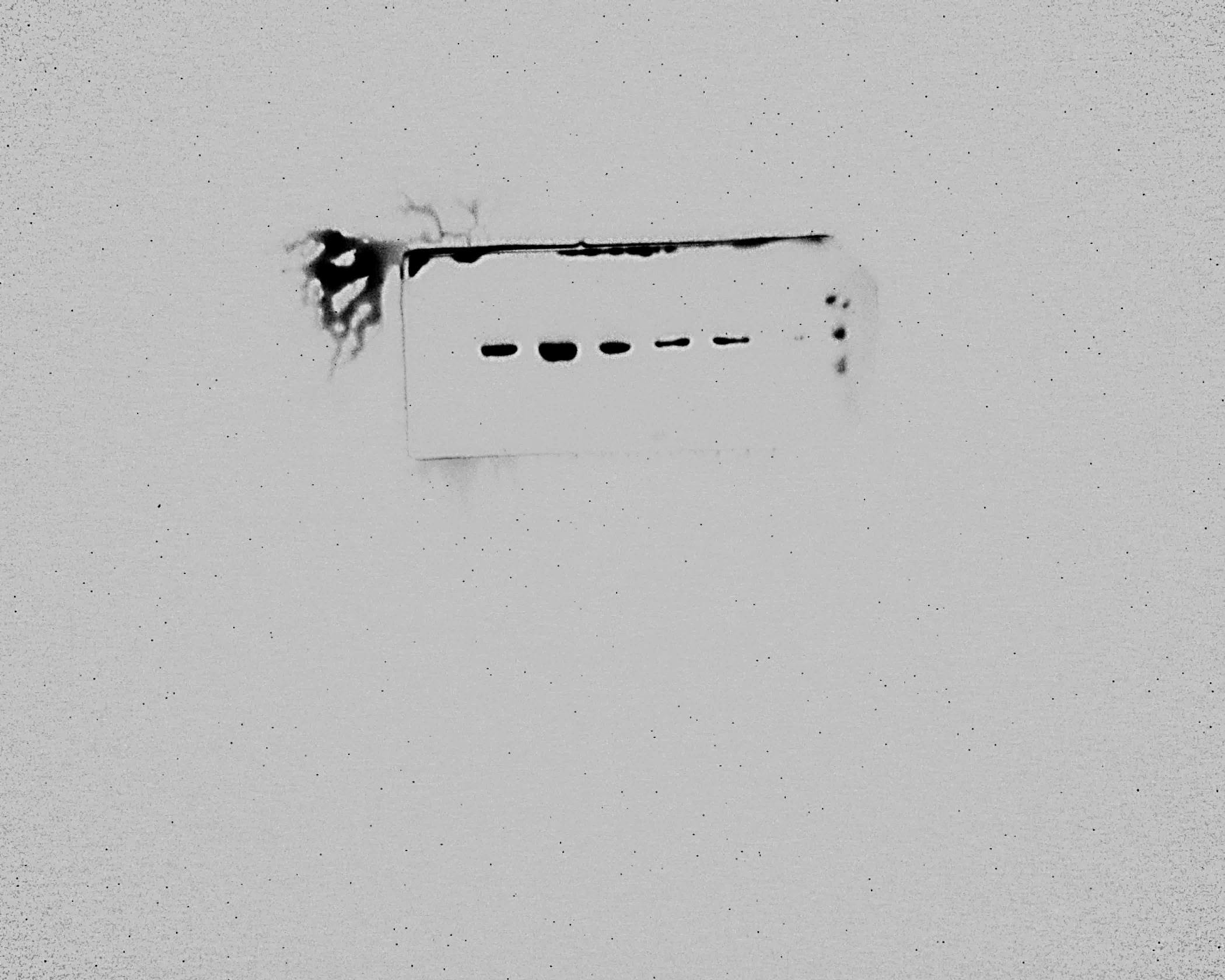
**

NLRP3

Control: C

Phosphate: P_i_

Ceramide: CER

Disulfiram: D

**Figure 10**


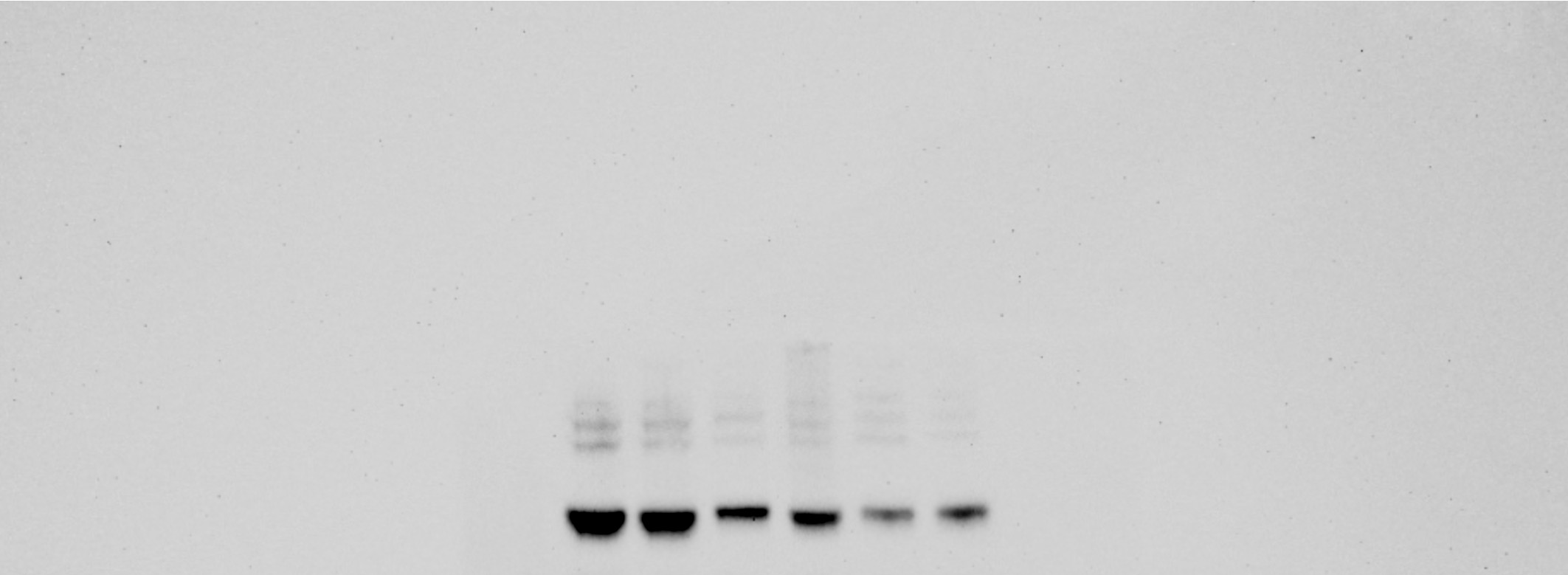

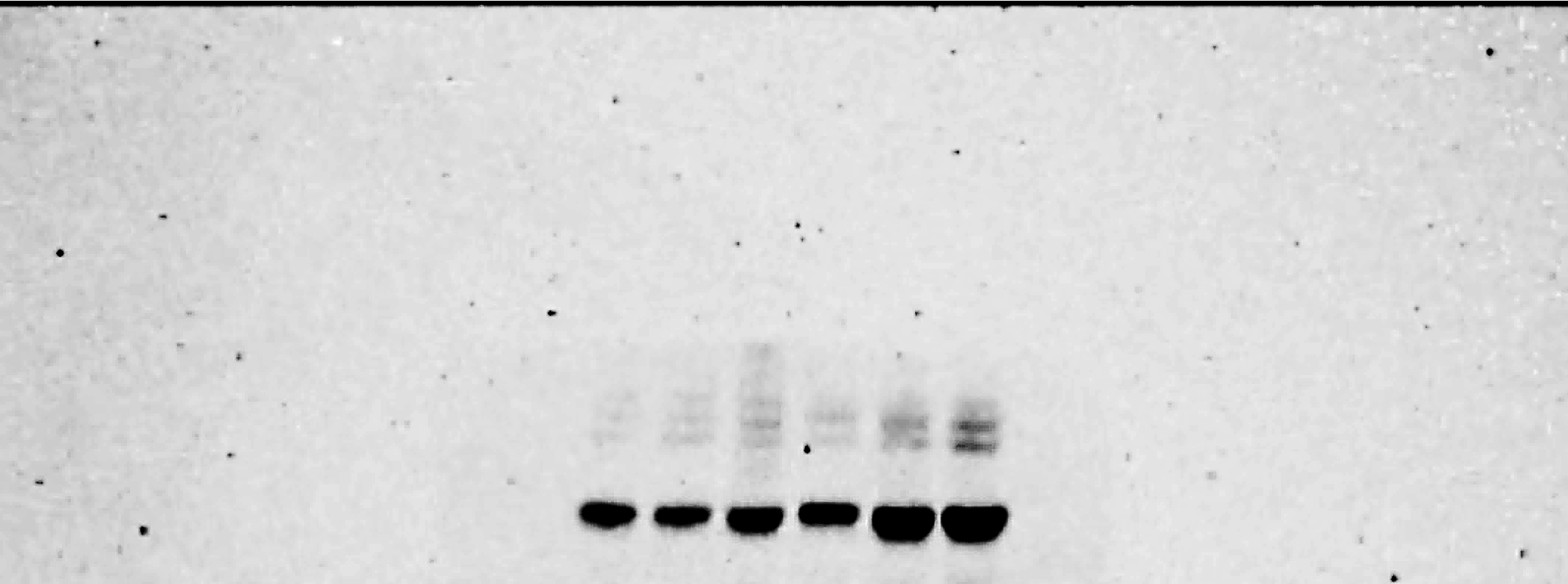

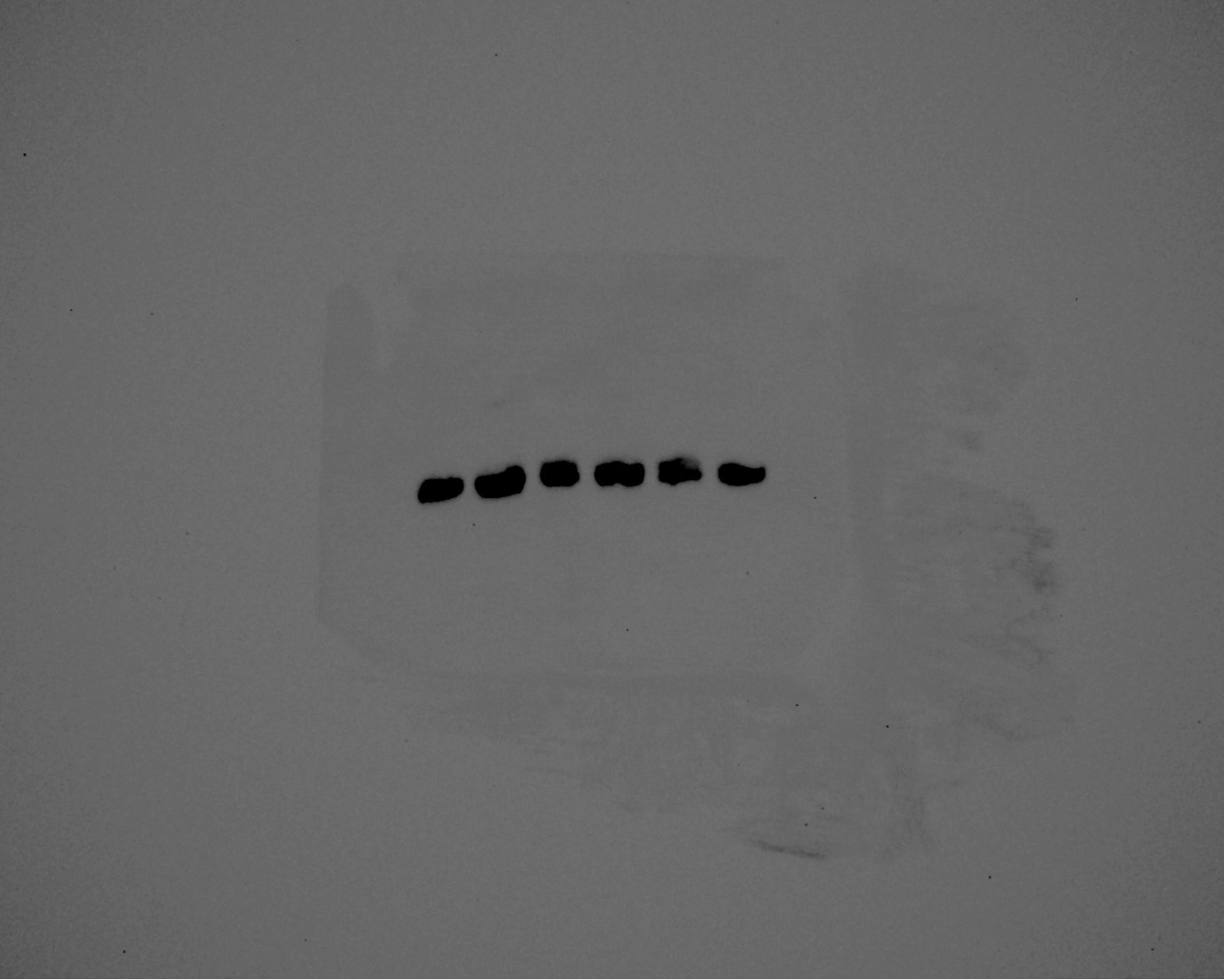


Control: C

Phosphate: P_i_

Evogliptin: E

Ceramide: CER

RUNX2

SM22α

GAPDH

**C P_i_  E+P_i_ CER CER +P_i_ E+CER+P_i_**

**Figure 11**

**
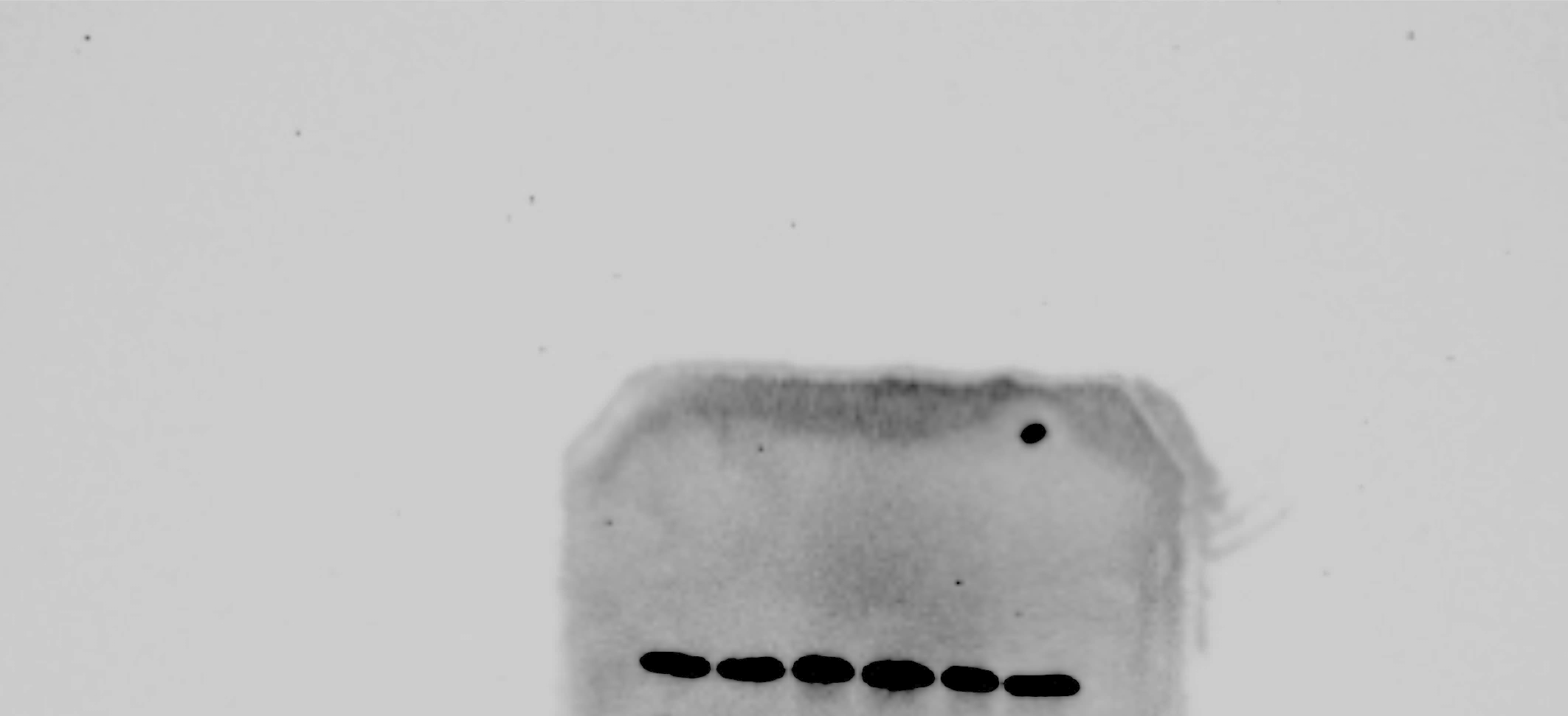
**

**C P_i_  E+P_i_ CER CER +P_i_ E+CER+P_i_**

**
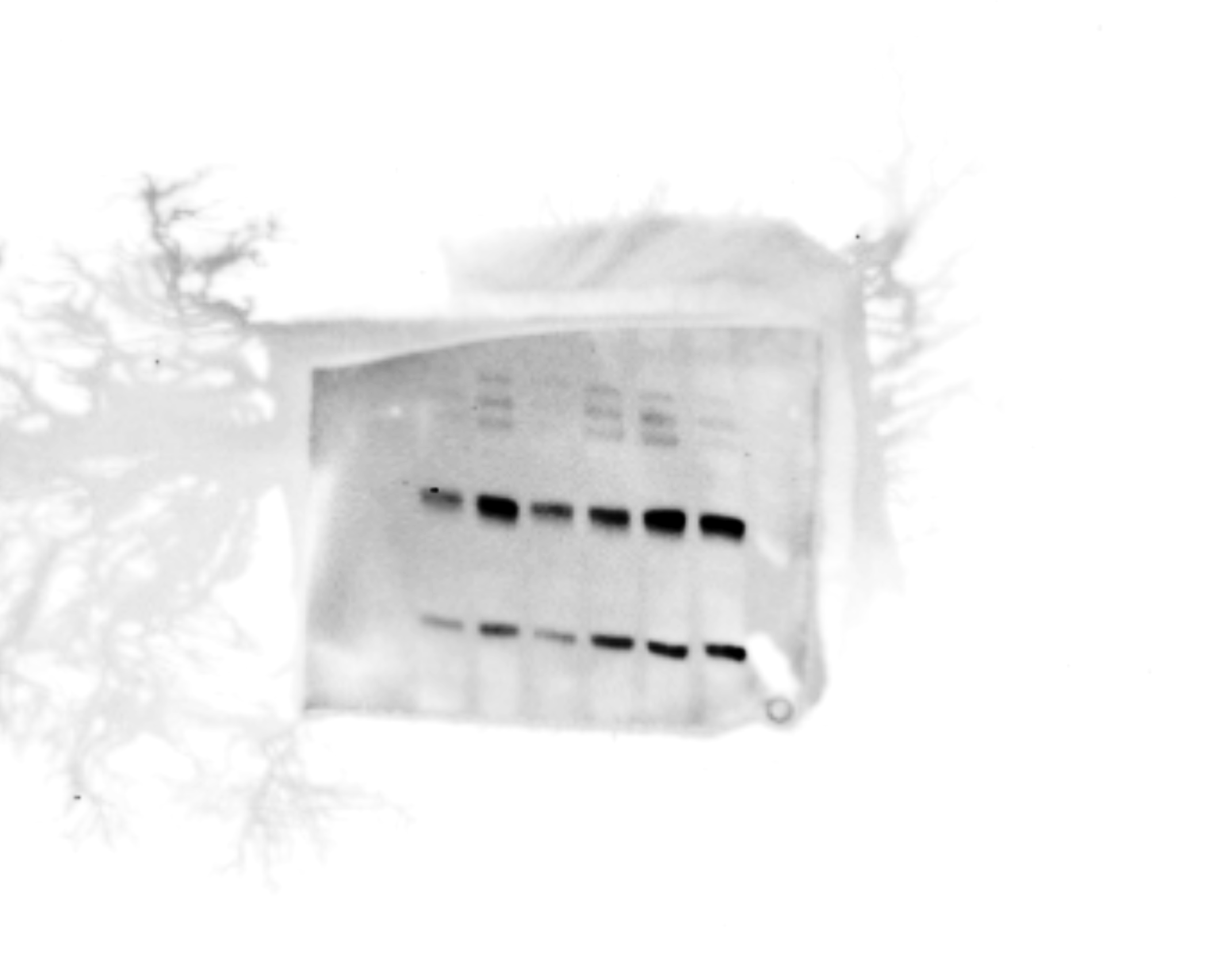

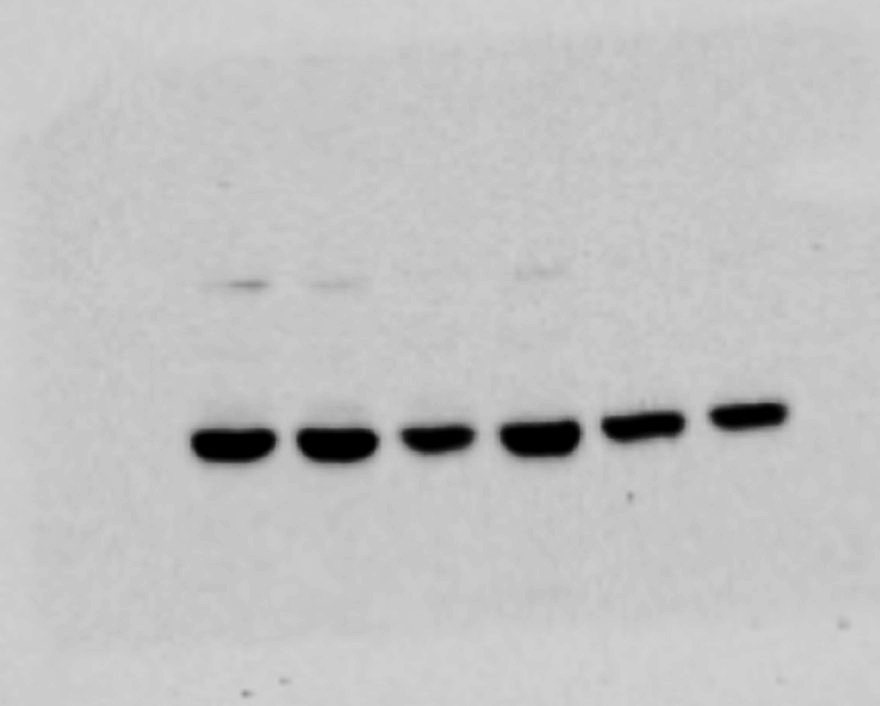

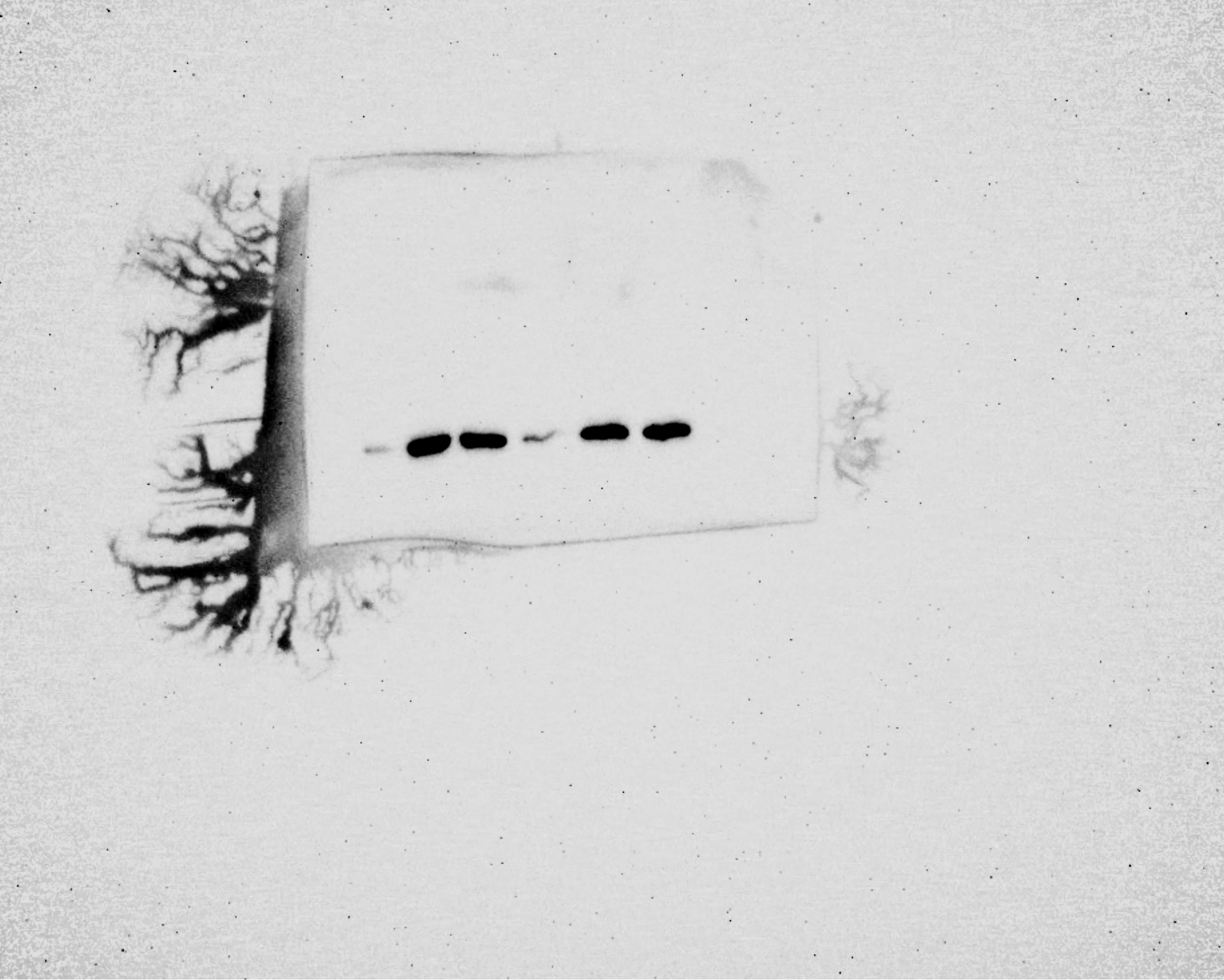
**

Control: C

Phosphate: P_i_

Evogliptin: E

Ceramide: CER

GSDM-D cleavage and activation

NLRP3

GSDM-D

GAPDH
